# Transconjugant range of PromA plasmids in microbial communities is predicted by sequence similarity with the bacterial host chromosome

**DOI:** 10.1099/mgen.0.001043

**Published:** 2023-06-21

**Authors:** Maho Tokuda, Masahiro Yuki, Moriya Ohkuma, Kazuhide Kimbara, Haruo Suzuki, Masaki Shintani

**Affiliations:** ^1^​ Department of Environment and Energy Systems, Graduate School of Science and Technology, Shizuoka University, 3-5-1, Johoku, Naka-ku, Hamamatsu, Shizuoka 432-8561, Japan; ^2^​ Japan Collection of Microorganisms, RIKEN BioResource Research Center, 3-1-1, Koyadai, Tsukuba, Ibaraki, 305-0074, Japan; ^3^​ Faculty of Environment and Information Studies, Keio University, 5322 Endo, Fujisawa-shi, Kanagawa 252-0882, Japan; ^4^​ Research Institute of Green Science and Technology, Shizuoka University, 3-5-1, Johoku, Naka-ku, Hamamatsu, Shizuoka, 432-8561, Japan

**Keywords:** Plasmid, host range, PromA, nucleotide compositions, conjugation

## Abstract

Nucleotide sequence similarity, including *k*-mer plasmid composition, has been used for prediction of plasmid evolutionary host range, representing the hosts in which a plasmid has replicated at some point during its evolutionary history. However, the relationships between the bacterial taxa of experimentally identified transconjugants and the predicted evolutionary host ranges are poorly understood. Here, four different PromA group plasmids showing different *k*-mer compositions were used as model plasmids. Filter mating assays were performed with a donor harbouring plasmids and recipients of bacterial communities extracted from environmental samples. A broad range of transconjugants was obtained with different bacterial taxa. A calculation of the dissimilarities in *k*-mer compositions as Mahalanobis distance between the plasmid and its sequenced transconjugant chromosomes revealed that each plasmid and transconjugant were significantly more similar than the plasmid and other non-transconjugant chromosomes. These results indicate that plasmids with different *k*-mer compositions clearly have different host ranges to which the plasmid will be transferred and replicated. The similarity of the nucleotide compositions could be used for predicting not only the plasmid evolutionary host range but also future host ranges.

## Data Summary

The authors confirm that all supporting data, codes, and protocols have been provided within the article or through supplementary data files.

1. The genome data obtained in this study are available in the DDBJ under accession numbers LC716920 to LC717408 for 16S rRNA genes. Amplicon sequences are in the DDBJ Sequence Read Archive (DRA) with accession number DRA014770 (BioSample IDs are SAMD00510300–SAMD00510329), and the results (read numbers) are provided in the supplementary data file (Table S6, available in the online version of this article). The raw sequence data of 107 transconjugants were also submitted to the DRA under accession number DRA012028. The BioSample ID are presented in Table S8. Their assembled sequences are available in Figshare (https://doi.org/10.6084/m9.figshare.20747521.v1) [1].
2. A total of 2772 prokaryotic complete chromosomes were obtained as reference genomes from the NCBI genome list (prok_reference_genomes.txt in the Refseq database https://www.ncbi.nlm.nih.gov/genome/) on 14 January 2021. The information is presented in Table S7.
3. The DDBJ/GenBank accession numbers of PromA plasmids are listed in Table S2.
4. The R code used for analysis and figure generation is accessible at Github (https://github.com/tokudamaho/Tokuda_et_al._PromA_plasmid).

**Impact Statement**

Plasmids can promote bacterial evolution and adaptation through conjugative transfer. Therefore, the bacterial range in which a plasmid can be transferred and replicated is an important feature of a plasmid (transconjugant range). However, it is challenging to comprehensively elucidate the transconjugant range of different plasmids using experimental mating assays. The evolutionary host range can be predicted using the *k*-mer plasmid compositions and bacterial chromosomes. Conversely, the bacterial transconjugant range has not been predicted based on nucleotide sequences. This study demonstrated that bacterial taxa of plasmid transconjugants with different *k*-mer compositions were different, even though the plasmids belonged to the same incompatibility group. The *k*-mer compositions between the plasmids and those of the chromosomes of each transconjugant were more similar than those between the plasmid and most bacterial chromosomes. Our findings show that the host ranges of plasmids could be partially predicted based on the nucleotide sequences of plasmids and bacterial chromosomes. This study provides an important clue for tracking gene transfer via plasmid conjugation in nature.

## Introduction

Plasmids are mobile genetic elements that are transferred among diverse bacteria [2]. Plasmid transfer can promote rapid evolution and adaptation to the natural environment because they generally carry accessory genes involved in antibiotic and heavy metal resistance and/or metabolism of various compounds. Plasmids are major contributors to the occurrence and spread of multidrug-resistant bacteria, posing a global public health threat [3]. Therefore, the host ranges – the types of bacteria to which plasmids can be transferred by conjugation and replicated in – provide important information on how they can promote bacterial evolution and adaptation and transmit resistance genes. The plasmid host range is defined in three different ways, replication, transfer, and evolutionary host ranges. The replication host range refers to the bacteria in which a plasmid can replicate, whereas the transfer host range refers to the bacteria to which a plasmid can be transferred (not necessarily replicated in the cell). Thus, the transfer host range is generally broader than the replication host range [4]. These plasmid host ranges are determined using filter and/or liquid mating assays between donor strain(s) and recipients (transconjugant ranges). Several studies have comprehensively identified the transconjugants of plasmids including members of IncP/P-1 [5–12], IncP-7 [13], IncP-9 [13], IncH1A [14], IncQ/P-4 [15], PromA (only PromAβ−1) [16], and pSN1216-29 [17]. However, the number of plasmids whose host ranges have been analysed is still limited because the analyses are time-consuming and costly, and it is unfeasible to experimentally identify all plasmid transconjugant ranges. Conversely, the evolutionary host range represents the hosts in which a plasmid has replicated at some point during its evolutionary history, predicted by using *k*-mer compositions but not proved experimentally [18, 19].

Owing to advances in bacterial genome sequencing, the relationships between the plasmid nucleotide composition and its host chromosome have been revealed. The plasmid GC content is usually lower than that of the host chromosomes, and the differences between them are within 5–10 % [4, 20, 21]. Plasmid *k*-mer compositions, a substring of length *k* in DNA sequences, are similar to those of host chromosomes [18, 22] and can be used to discern whether the plasmid has a broad or narrow host range [19]. Additionally, *k*-mer compositions are also used to identify the putative evolutionary hosts of plasmids, in which a plasmid has been replicated at some point during its evolutionary history [23]. However, few studies have compared the bacterial taxa of experimentally identified transconjugants (transconjugant range) with the predicted evolutionary host ranges [17, 24]. Although the transconjugant range is important for understanding plasmid behaviour in the environment, it is still unclear whether transconjugant ranges can be predicted based on nucleotide sequences of plasmids and bacterial chromosomes.

PromA is a recently proposed plasmid group that includes broad-host-range plasmids [25]. To date, 41 PromA plasmids have been reported and completely sequenced [25–30]. Four PromA plasmids belonging to three different subgroups [pSN1104-11 (PromAγ), pMH0613-68 (PromAβ−1), pYK0414-12 (PromAβ−2), and pSN0729-62 (PromAδ)] were isolated from different environmental samples [29, 30]. Their GC contents showed up to 10 % difference [63.7 % for pSN1104-11 (PromAγ), 60.7 % for pMH0613-68 (PromAβ−1), 56.5 % for pYK0414-12 (PromAβ−2), and 54.2 % for pSN0729-62 (PromAδ)]. Therefore, the evolutionary host range of these PromA plasmids may be different. Here, transconjugant ranges of PromA plasmids with different nucleotide compositions were compared using a mating assay. *In silico* analyses were performed to compare the dissimilarity in *k*-mer compositions between plasmids and transconjugant chromosomes obtained through experimental mating assays.

## Methods

### Bacterial strains, plasmids, and culture conditions

The bacterial strains and plasmids used here are listed in Table 1. The bacterial strains were grown in Luria broth (LB) [31] at 30 °C for *Pseudomonas* and 37 °C for *Escherichia *. Antibiotics were used at final concentrations of 50 µg ml^−1^ kanamycin (Km), 50 µg ml^−1^ tetracycline (Tc), and 50 µg ml^−1^ rifampicin (Rif). Cycloheximide was added at 100 µg ml^−1^ to prevent the growth of fungi when microbial communities from the environmental samples were cultured. The solid media were prepared by the addition of 1.5 % (w/v, final concentration) agar to LB agar plates. The agar plate without any nutrients was prepared by mixing dH_2_O with 1.5 % (w/v, final concentration) agar, named the Agar plate.

**Table 1.** Bacterial strains and plasmids used in this study

Strain or plasmid	Relevant characteristics	Reference
**Bacterial strains**		
**Escherichia coli**		
JM109	F' *traD36 proA*+*proB*+*lacI* ^*q*^ Δ(*lacZ*)M15 Δ(*lac-proAB*) *supE44 hsdR17 recA1 gyrA96 thi-1 endA1 relA1* e14- λ^-^	RBC Bioscience
S17-1 λpir	RK2 *tra* regulon; host for *pir* ^*-*^dependent plasmids; recA *thi pro hsdRM* RP4-2-Tc:Mu-Km:Tn7*λpir* Tp^r^ Sm^r^	[34]
		
Pseudomonas putida		
KT2440	pWW0-free Pseudomonas putida mt-2	[55]
KT2440 (pBBR1MCS-2_11*repA*)	KT2440 harbouring pBBR1MCS-2_11*repA*	This study
KT2440 (pBBR1MCS-2_68*repA*)	KT2440 harbouring pBBR1MCS-2_68*repA*	This study
KT2440 (pBBR1MCS-2_12*repA*)	KT2440 harbouring pBBR1MCS-2_12*repA*	This study
KT2440 (pBBR1MCS-2_62*repA*)	KT2440 harbouring pBBR1MCS-2_62*repA*	This study
SMDBS	A *dapB*-deleted strain of SM1443, Rif^r^ of KT2440 (KT2442) with mini-Tn*5*-*lacI* ^*q*^ cassette inserted into the chromosome	[13]
SMDBS (pSN1104-11::*gfp*)	SMDBS harbouring pSN1104-11::*gfp*	This study
SMDBS (pMH0613-68::*gfp*)	SMDBS harbouring pMH0613-68::*gfp*	This study
SMDBS (pYK0414-12::*gfp*)	SMDBS harbouring pYK0414-12::*gfp*	This study
SMDBS (pSN0729-62::*gfp*)	SMDBS harbouring pSN0729-62::*gfp*	[30]
		
Pseudomonas resinovorans		
CA10dm4R	Derivative strain of CA10dm4 spontaneously Rif^r^.	[56]
CA10dm4RG	Derivative strain of CA10dm4 spontaneously Rif^r^ with introduced Gm^r^ gene.	[56]
CA10dm4RGFP	CA10dm4R (spontaneous rifampicin-resistant CA10dm4), miniTn*7*(Gm) P_*A1/O4/O3*_-gfp-a was inserted into the chromosome (Gm^r^, Cm^r^).	[29]
CA10dm4RGFP(pSN1104-11, pBBR1MCS-2)	CA10dm4RGFP harbouring pSN1104-11 and pBBR1MCS-2	[29]
CA10dm4RGFP (pMH0613-68, pBBR1MCS-2)	CA10dm4RGFP harbouring pMH0613-68 and pBBR1MCS-2	[30]
CA10dm4RGFP (pYK0414-12, pBBR1MCS-2)	CA10dm4RGFP harbouring pYK0414-12 and pBBR1MCS-2	[30]
Plasmids		
pJBA28	Ap^r^, Km^r^, delivery plasmid for mini-Tn*5*-Km-P_*A1/04/03*_-RBSII-gfpmut3*-T_0_-T_1_	[33]
pSN1104-11	PromAγ conjugation plasmid	[29]
pMH0613-68	PromAβ−1 conjugation plasmid	[30]
pYK0414-12	PromAβ−2 conjugation plasmid	[30]
pSN0729-62	PromAδ conjugation plasmid	[29]
pBBR1MCS-2	Km^r^, *lacZα mob;* compatible with IncP, IncQ, and IncW plasmids	[32]
pBBR1MCS-2_11*repA*	1,623 bp DNA region containing *repA* of pSN1104-11 cloned at EcoRI site of pBBR1MCS-2	This study
pBBR1MCS-2_68*repA*	1,612 bp DNA region containing *repA* of pMH0613-68 cloned at EcoRI site of pBBR1MCS-2	This study
pBBR1MCS-2_12*repA*	1,423 bp DNA region containing *repA* of pYK0414-12 cloned at EcoRI site of pBBR1MCS-2	This study
pBBR1MCS-2_62*repA*	1,440 bp DNA region containing *repA* of pSN0729-62 cloned at EcoRI site of pBBR1MCS-2	This study
pUC19	Ap^r^; E. coli cloning vector	[57]
pUC19_11*oriV*Tc	2,057 bp DNA region containing *oriV* of pSN1104-11 and Tc^r^ gene of pBBR1MCS-3 cloned at EcoRI site of pUC19	This study
pUC19_68*oriV*Tc	1,874 bp DNA region containing *oriV* of pMH0613-68 and Tc^r^ gene of pBBR1MCS-3 cloned at EcoRI site of pUC19	This study
pUC19_12*oriV*Tc	1,970 bp DNA region containing *oriV* of pYK0414-12 and Tc^r^ gene of pBBR1MCS-3 cloned at EcoRI site of pUC19	This study
pUC19_62*oriV*Tc	2,043 bp DNA region containing *oriV* of pSN0729-62 and Tc^r^ gene of pBBR1MCS-3 cloned at EcoRI site of pUC19	This study
pSN1104-11::*gfp*	Km^r^-P_*A1/O4/O3*_-RBSII-gfpmut3*-T_0_-Cm^r^-T_1_ cassette was inserted in 38 362 nt (ORF_046) of pSN1104-11	This study
pMH0613-68::*gfp*	Km^r^-P_*A1/O4/O3*_-RBSII-gfpmut3*-T_0_-Cm^r^-T_1_ cassette was inserted in 40 893 nt (ORF_051) of pMH0613-68	This study
pYK0414-12::*gfp*	Km^r^-P_*A1/O4/O3*_-RBSII-gfpmut3*-T_0_-Cm^r^-T_1_ cassette was inserted in 35 137 nt (non-coding region) of pYK0414-12	This study
pSN0729-62::*gfp*	Km^r^-P_*A1/O4/O3*_-RBSII-gfpmut3*-T_0_-Cm^r^-T_1_ cassette was inserted in 35 612 nt (non-coding region) of pSN0729-62	[30]

### Standard DNA manipulations

The total DNA of the bacterial cells was extracted using the NucleoSpin Tissue Kit or NucleoSpin 96 Tissue (Macherey-Nagel, Duren, Germany). DNA from transconjugant communities and environmental samples (see below) was extracted using the DNeasy PowerSoil Kit (GmbH; Qiagen, Hilden, Germany). Polymerase chain reaction (PCR) was performed on a T100 thermal cycler (Bio-Rad Laboratories, Hercules, CA, USA) with the primer and KOD One Master Mix (Toyobo Co., Ltd., Osaka, Japan). PCR product clean-up was performed using ExoSAP-IT Express (Thermo Fisher Scientific, Waltham MA, USA).

### Functional analyses of RepA and *oriV* regions of PromA plasmids

The *repA* encoding replication initiation protein and *oriV* (origin of replication) regions of pSN1104-11, pMH0613-68, pYK0414-12, and pSN0729-62 were amplified using PCR and the primer set listed in Table S1. Each *repA* gene was cloned into a broad-host-range vector pBBRMCS-2 [32], whereas each *oriV* region was cloned into pUC19 using the NEB HiFi DNA Assembly Master Mix (New England Biolabs, Ipswich, MA, USA). To assess whether each *repA* gene product could function on the *oriV* of different PromA plasmids, *Pseudomonas putida* KT2440, harbouring one of the plasmids containing the *repA* gene under control of the lac promoter (pBBR1MCS-2_11*repA*, pBBR1MCS-2_68*repA*, pBBR1MCS-2_12*repA*, and pBBR1MCS-2_62*repA*), was transformed with the plasmid containing one of the *oriV* of PromA plasmids (pUC19_11*oriV*Tc, pUC19_68*oriV*Tc, pUC19_12*oriV*Tc, or pUC19_62*oriV*Tc). Transformation was performed as follows: *P. putida* KT2440 (pBBR1MCS-2 with *repA* genes) was cultured until OD_600_=0.75 with Km. After harvesting the cells, they were washed by dH_2_O twice, then resuspended in 10 % glycerol (competent cell). Two microlitre plasmid DNA of pUC19 containing the *oriV* region of each PromA plasmid was added to the competent cells. Electroporation was performed with 1.8 kV pulse using a MicroPulser (Bio-Rad Laboratories). The transformation frequency was calculated by dividing the number of colonies (c.f.u.) by amount of plasmid DNA (micrograms). pUC19 without any additional DNAs was used as a negative control as it is not able to replicate in *P *. *putida *. When the transformants were detected on LB +Km+ Tc plates, the *repA* product was considered functional on *oriV*.

### Construction of *gfp*-tagged plasmids

The four PromA plasmids (pSN1104-11, pSN0729-62 [30], pMH0613-68, and pYK0414-12) were transferred to *P *. *resinovorans* CA10rm4R fromCA10dm4RGFP with pBBRMCS-2 [30]. Then mini-Tn*5* (3 859 bp) with P*_A1/O4/O3_*-RBSII-gfpmut3* and the Km-resistance gene was randomly inserted into the four PromA plasmids using pJBA28 [33] and *E. coli* S17-1 λ*pir* [34] as described previously [17]. The mini-Tn*5* was inserted into 38 362 nucleotides (nt) of pSN1104-11, 40 891 nt of pMH0613-68, 35 137 nt of pYK0414-12, and 35 612 nt of pSN0729-62. Both disrupted genes of pSN1104-11 and pMH0613-68 were hypothetical genes and mini-Tn*5* were inserted into non-coding regions for pYK0414-12 and pSN0729-62 (Table 1). As their randomly inserted region was not expected to affect the basic function of the plasmids, these GFP-tagged plasmids were used for further analyses.

### Comparisons of the transconjugants ranges of each PromA plasmid

The donor strain, *P. putida* SMDBS, harbouring *gfp*-tagged PromA plasmids, was cultured in LB with Km. Model microbial communities in environmental samples (soil and lake water) were used as recipient bacteria. Soil samples were collected at Shizuoka University, Hamamatsu, Japan (34.73 N 137.72E), on 17 July 2020 and (34.72 N 137.72E) on 12 August 2020. Water was collected from Lake Sanaru, Hamamatsu, Japan (34.72 N 137.70E) on 12 August 2020. Extraction of the microbial fraction from the environmental samples was performed using Histodenz (Sigma-Aldrich, St. Louis, MO, USA) as described previously [13]. The number of bacterial cells of extracted fraction and donor culture was counted by microscopy (BZ-X700, KEYENCE CORP.) after staining with SYBR Green I Nucleic Acid Gel Stain (TaKaRa BIO Inc.) according to the manufacturer’s instruction. Overnight cultured donors were harvested, washed with phosphate-buffered saline (PBS), and resuspended in 100 µl PBS or LB. The donor (10^8^–10^9^ cells) and recipient (10^8^–10^9^ cells, microbial communities extracted from soil and lake water) mixture was added to 0.45 µm pore-size filters on different plates. The mating assays were performed under three different conditions, (i) ‘Soil-LBAgar’ mating on LB agar plates for 2 h and then transferred to Agar plates for 48 h with recipients extracted from the soil sample (July); (ii) ‘Soil-Agar’ mating on Agar plates for 48 h with recipients extracted from the soil sample (August); (iii) ‘Lake-Sanaru-LBAgar’ mating on LB agar plates for 2 h and then transferred to Agar plates for 48 h with recipients extracted from the lake sample. Detection and separation of transconjugant cells were performed using flow cytometry and the cell sorter MoFlo XDP IntelliSort II instrument (Beckman Coulter, Brea, CA, USA) equipped with a CyClone robotic arm for plate sorting (384 well sorting mode) using a 488 nm argon laser and a 70 -µm nozzle orifice. Sorting of each transconjugant cell was performed as previously described [13]. Each of the 384 cells was sorted on LB plates and incubated at 30 °C for 2 d to allow the cells to form colonies [the culture-dependent (CD) method]. For the culture-independent (CI) method, 15 000 transconjugant cells in the soil microbes were sorted into 100 µl PBS in a microtube, and their DNA was extracted (see above).

### Identification of transconjugants

Identification of transconjugants obtained using the CD method was performed by sequencing a partial region of the 16S rRNA gene (V3/V4) using the 805R primer (5′-GACTACCAGGGTATCTAATC-3′) amplified with 27F (5′-AGAGTTTGATCMTGGCTCAG-3′) and 1492R (5′-TACGGYTACCTTGTTACGACTT-3′) using KOD One (Toyobo). The conditions were as follows: 30 cycles at 98 °C for 10 s, 55 °C for 5 s, 68 °C for 5 s, and then held at 12 °C. Partial nucleotides of the PCR products were sequenced by the Sanger method using the 805R primer. The presence of plasmids in the transconjugants was confirmed by PCR of the DNA region (*repA* and *traS*) in each plasmid using the primer sets shown in Table S1. The conditions were as follows: 30 cycles at 98 °C for 10 s, 55 °C for 5 s, 68 °C for 1 s, and then held at 12 °C. The amplified products were subjected to agarose gel electrophoresis and their sizes were confirmed.

**Fig. 1.**
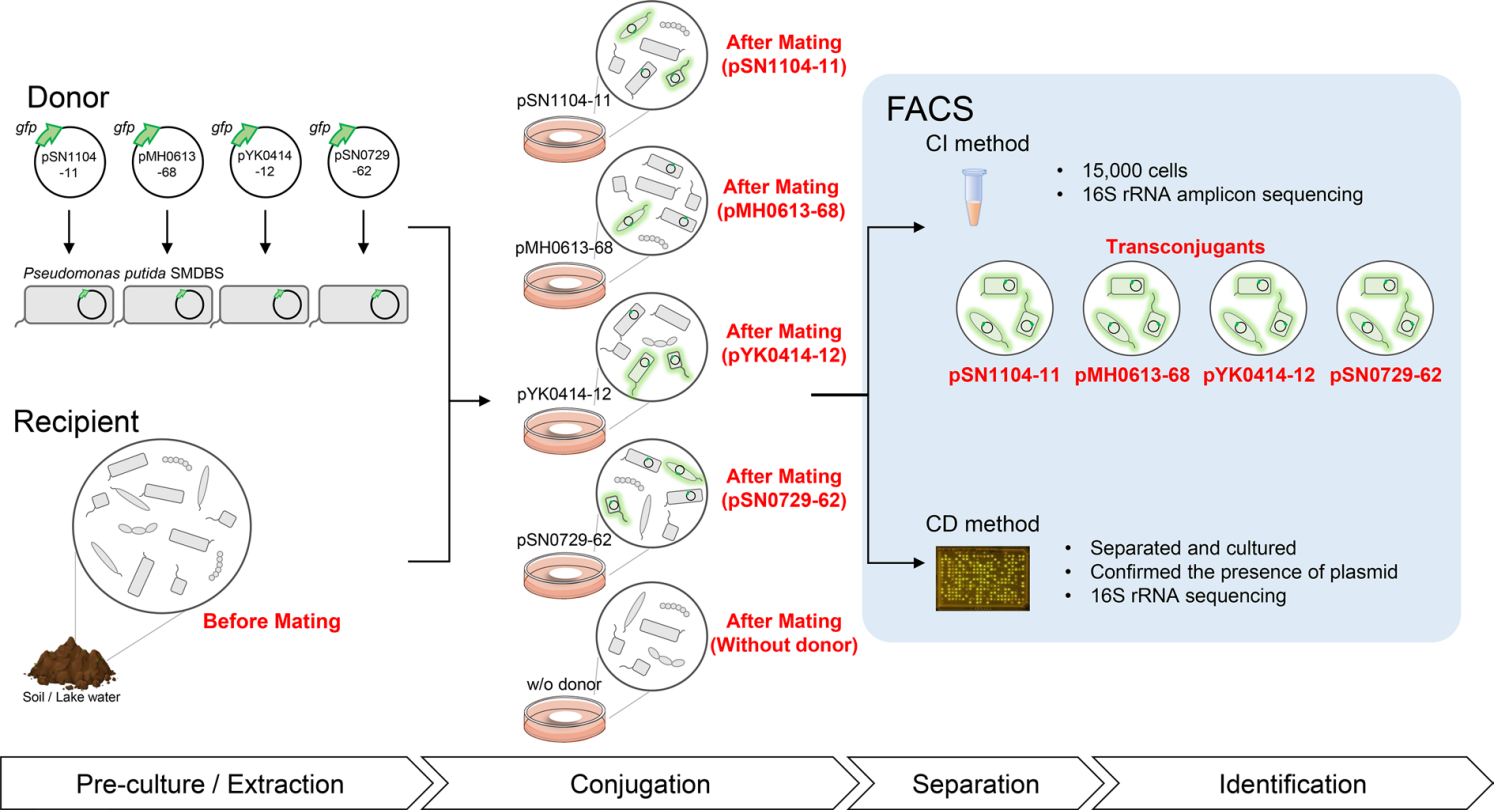
Procedures of the mating assay with four PromA plasmids and microbial communities showing the types of samples analysed using the CI method (red). These mating assays were performed using three mating conditions, Soil-LBAgar, Soil-Agar, and Lake-Sanaru-LBAgar as described in the Methods.

To compare the changes in microbial communities, amplicon sequencing was performed for three types of samples: ‘Before Mating’ (microbial community extracted from soil and lake water), ‘After Mating’ (the community just before sorting) and ‘Transconjugants’ (isolated green-fluorescent cells by the CI method) (Fig. 1). In each mating assay, there was one sample of ‘Before Mating’, five [four plasmids and one sample without (w/o) donor] of ‘After Mating’ and four (plasmids) of ‘Transconjugants’ for sequencing (Fig. 1). Regarding ‘After Mating w/o donor’, microbial communities (from soil and lake water) were incubated under the same conditions as the mating assay. The 16S rRNA gene (V4) amplicon sequencing was performed as follows. The first PCR was performed with the primer sets 515f-MIX (5′-ACACTCTTTCCCTACACGACGCTCTT CCGATCTNNNNNGTGCCAGCMGCCGCGGTAA-3′) and 806 r_MIX (5′-GTGACTGGAGTTCAGACGTGTGCTCTTC CGATCTNNNNNGGACTACHVGGGTWTCTAAT-3′) using ExTaq HS (TAKARA BIO, Shiga, Japan). This was set up at 94 °C for 2 min, followed by 30 cycles of 94 °C for 30 s, 50 °C for 30 s, 72 °C for 30 s, and 72 °C for 5 min. After purification of the PCR products, a second PCR was performed with the primer set 2ndF (5′-AATGATACGGCGACCACCGAGATCTACAC-Index2-ACACT CTTTCCCTACACGACGC-3′) and 2ndR (5′-CAAGCAGAA GACGGCATACGAGAT-Index1-GTGACTGGAGTTCAGACG TGTG-3′) using ExTaq HS (TAKARA BIO). Nucleotide sequences were determined using MiSeq (2×300 bp, Illumina San Diego, CA, USA). The read sequences matching the primer sequences were extracted using the barcode splitter of the FASTX-Toolkit [35] and the reads were trimmed with a quality threshold >20 using a sickle [36]. All sequencing reads shorter than 40 bp were excluded from the analysis. The reads were merged using FLASH software with a minimum overlap of 10 bp [37]. The 246–260 base reads and the above 16S rRNA gene sequences of the CD method were used for transconjugant identification using Geneious Prime 2020 software [38] with the sequences in NCBI serving as the reference. The sequences of the 16S rRNA transconjugant genes obtained by the CD method were deposited in the DDBJ under accession numbers LC716920 to LC717408 (482 sequences, seven of which were plasmid negative strains). The amplicon sequence data were deposited in the DRA under accession numbers DRA014770 (BioSample IDs are SAMD00510300–SAMD00510329). The raw sequence read data of 107 transconjugants were also deposited in the DRA with accession number DRA012028 (each BioSample ID is listed in Table S8).

### Bioinformatics and statistical analyses

Data analysis, calculation of *k*-mer compositions, and creation of clustered heatmaps were implemented using R software version 4.1.2 [39]. The differences in microbial communities between samples (beta diversity) were measured using the weighted UniFrac distance [40] calculated using the ‘GUniFrac’ package version 1.1 of R [41]. Principal coordinate analysis (PCoA) was performed using the distance matrix (weighted UniFrac distances). The ‘phyloseq’ package version 1.34.0 of R was used for constructing the PCoA plot to visualise the inter-sample microbiome difference in two-dimensional space. Heatmaps were drawn using the ‘ComplexHeatmap’ package version 2.14.0 of R [42].

The *k*-mer compositions of the PromA plasmids were compared with those of bacterial chromosomes. Here, three-mer compositions were used to calculate the dissimilarity of plasmids and bacterial chromosomes as it was the best to distinguish between broad- and narrow-host-range plasmids and to predict their evolutionary host candidates, as in prior studies [18, 19]. Two- and four-mer are shown in the supplemental material for each analysis. A total of 2774 prokaryotic complete chromosomes were obtained as reference genomes from the NCBI genome list (prok_reference_genomes.txt in the Refseq database https://www.ncbi.nlm.nih.gov/genome/) on 14 January 2021. If the bacterial strain had multiple chromosomes, only the largest chromosome was used for analysis, as described previously [17]. Among the 2774 genomes, two (NZ_LR214974.1 and NZ_LR134410.1) had nucleotide sequences less than 320 kb, which were too short to compute the inverse matrix for the dissimilarity calculation (Mahalanobis distance, see below); thus, they were removed from the analyses. Reference genome sequence statistics, including length and GC content, are shown in Table S7.

The DNA of 107 transconjugants was extracted and sequenced using the Hiseq2500 platform (Illumina), and the obtained short reads were assembled using Unicycler v0.4.9 of Bactopia software [43]. The assembled contigs corresponding to the PromA plasmids were removed using nucleotide blast, and the resultant contigs were subjected to nucleotide composition analysis.

The *k*-mer compositions of the PromA plasmids were compared with the chromosomes of the 2772 references (complete genomes) and of 107 transconjugants (draft genomes) in the manner described previously [17, 19]. Briefly, the DNA words (2-, 3-, and 4-mer) were counted using both strands of a DNA sequence and normalised by background nucleotide composition. The values of *k*-mer compositions – the rho statistic with the odds ratio defined as the ratio of observed to expected values – were computed using the rho function of the ‘SeqinR’ package version 4.2.23 [44] The dissimilarity in *k*-mer compositions between an entire plasmid sequence and a set of non-overlapping 5 kb chromosomal segments from one bacterial strain was measured by using the Mahalanobis distance (*D*) in the equation below (the length of chromosomal segments, 5 kb, was selected because the median rank of the genomic signature similarities between plasmids and their host chromosome was highest among 2, 5, 10, and 20 kb fragments, as found in our previous study [18]).

$$D^{2}=(x-\mu {)}^{T}S^{-1}(x-\mu )$$

where x is a vector of *k*-mer abundance values for a plasmid, µ is the mean vector of *k*-mer abundance values calculated from the chromosomal segments, S is the variance-covariance matrix of the *k*-mer abundance values calculated from the chromosomal segments (S ^−1^ is the inverse matrix of S), and superscript T is the transposition operator. The *P*-value was also calculated based on the Mahalanobis distance squared, because *D* ^2^ has no upper limit. High *P*-values of close to one indicate a small *D* ^2^ and similar *k*-mer compositions between a plasmid and chromosome as described previously [17, 19].

## Results and discussion

### The four PromA plasmids have different nucleotide compositions

**Fig. 2.**
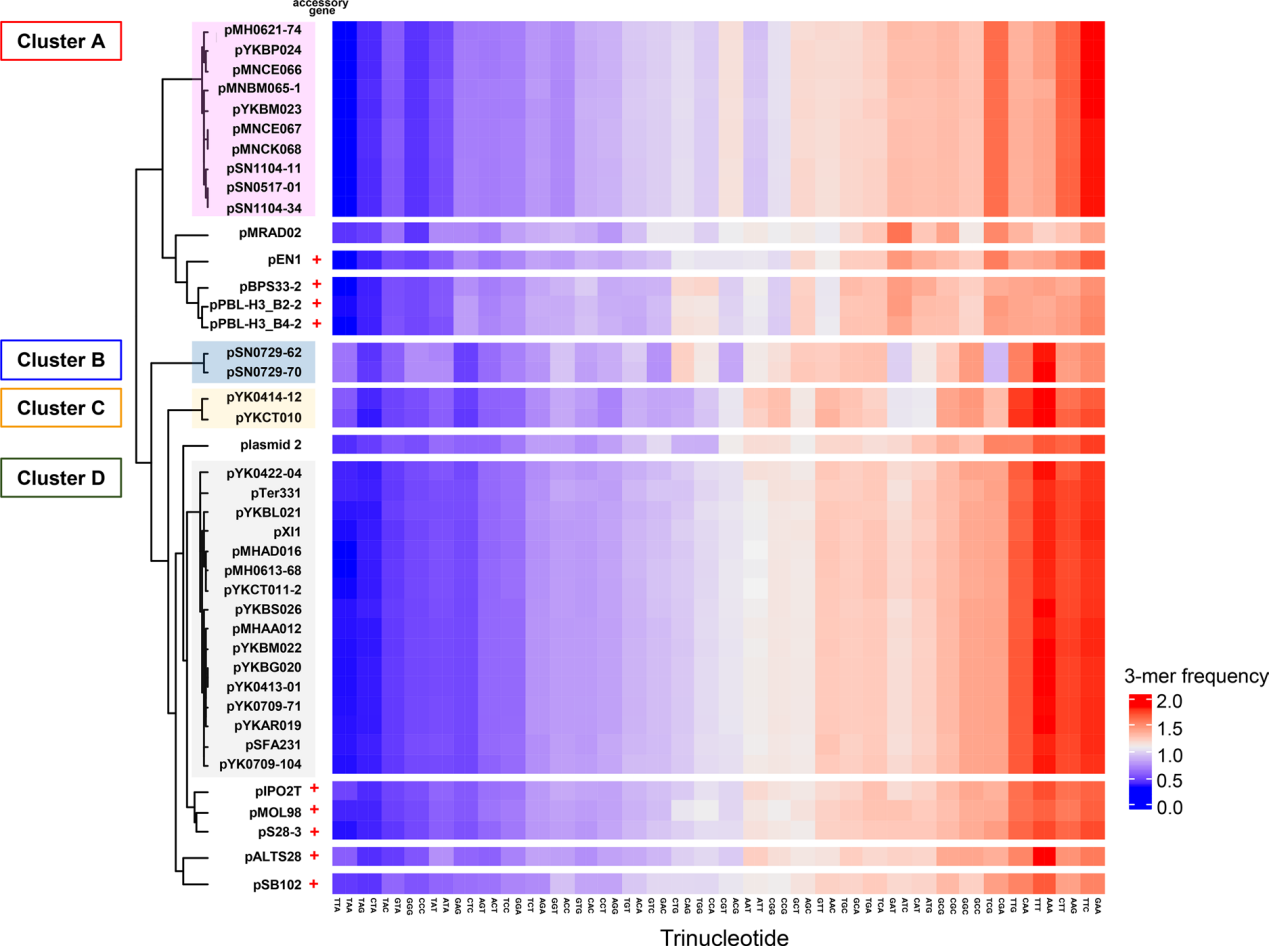
A clustered heatmap showing the dissimilarity of 3-mer compositions in PromA plasmids. Dissimilarities were calculated using Euclidean distance. Three-mer frequency is the observed 3-mer frequency divided by the expected 3-mer frequency. Nine plasmids carrying known accessory genes are indicated by ‘+’.

Clustering analyses of the *k*-mer (*k*=2, 3, 4) compositions of different PromA plasmids using Euclidean distance showed that they could be classified into several clusters (Figs 2 and S1). Currently, four subgroups (α, β, γ, and δ) are proposed for PromA group plasmids with two sub-subgroups (β−1 and β−2) based on phylogenetic analyses of their previously determined 24 conserved genes among 41 PromA plasmids [30]. For the 3-mer compositions, the four clusters were named A to D, which included ten (cluster A, pMH0621-74 to pSN1104-34), two (cluster B, pSN0729-62 and pSN0729-70), two (cluster C, pYK0414-12 and pYKCT010) and 16 plasmids (cluster D, pYK0422-04 to pYK0709-104). These coincided with the classifications of the sub (sub-sub) group of PromA plasmids (clusters A, B, C, and D for PromAγ, PromAδ, PromAβ−2, and PromAβ−1, respectively) [30] (Fig. 2). The PromAα plasmids [28], including pSB102 (carrying accessory genes, see below), plasmid two, and pMRAD02 (probably not a member of PromAα [30]) formed a cluster of their own (Fig. 2). As reported previously [29, 30], 32 among 41 PromA plasmids did not carry any previously known accessory genes, including antibiotic resistance and/or catabolic genes. The other nine plasmids – pSB102, pALTS28, pIPO2T, pS28-3, pMOL98, pPBL-H3_BS2-2, pPBL-H3_BS4-2, pBPS33-2, and pEN1 (Fig. 2) – carry genes involved in antibiotic/heavy metal resistance or linuron degradation [26, 27, 45–47]. Their DNA regions, including accessory genes, were 5.5 to 70 kb, and pBPS322-2, pPBL-H3_B4-1, and pPBL-H3_B2-2, which belong to PromAγ, contained more than 60 kb DNA regions. As the presence of such large DNA regions with accessory genes significantly affected the *k*-mer composition of the entire plasmid [48], these nine plasmids were found in a cluster different from cluster B, including the other PromAγ plasmids without any accessory genes (Fig. 2). With respect to the four clusters (A–D), they were also observed in the clustering analyses with 2- and 4-mer compositions (Fig. S1). Clustering analysis based on the GC content of 24 conserved genes also showed that each subgroup, except for PromAα (pSB102 and plasmid 2) and pMARD02, formed their own cluster as well as *k*-mer clustering (Fig. S2).

Hereafter, we focused on four plasmids without accessory genes: pSN1104-11 (PromAγ, Cluster A), pSN0729-62 (PromAδ, Cluster B), pMH0613-68 pYK0414-12 (PromAβ−2, Cluster C), and (PromAβ−1, Cluster D). Our previous studies have shown that the plasmids of different subgroups of PromA had different GC contents [29, 30]. Consistent with these results, we found that the GC content was 63.7 % for pSN1104-11, 54.2 % for pSN0729-62, 60.7 % for pMH0613-68, and 56.5 % for pYK0414-12. Notably, the GC content of the 24 genes conserved in all PromA plasmids [29] was always in the order of PromAγ, Cluster A>PromAβ−1, Cluster D>PromAβ−2, Cluster C>PromAδ, Cluster B (Fig. S2, Table S2), while there were several exceptions, *traD* in PromAβ−1 and PromAδ plasmids (shown in blue in Table S2) and a few others (shown in red in Table S2).

### The RepA and *oriV* regions between different subgroups of PromA plasmids are interchangeable

The *repA* gene and RepA protein of the four different PromA plasmids did not show high identities (less than 80%, Table S3), whereas the nucleotide sequences of putative RepA binding sites (iteron) were well conserved [29, 30]. Four different pUC19 vectors containing each *oriV* region of pSN1104-11, pMH0613-68, pYK0414-12, and pSN0729-62 were replicated in *P. putida* expressing RepA of pSN1104-11 from pBBR1MCS-2_11*repA*, while no transformants were detected with the pUC19 vector control (below the detection limit, Table S4). Similarly, the other RepA replicated vectors with different *oriV* regions of other PromA plasmids (Table S4). These results indicate that RepA can interchangeably bind and function as a replication initiation protein in each *oriV* region of different PromA plasmids. Therefore, these plasmids are incompatible with one another, as is the case for the IncP/P-1 plasmids [49–51]. This result also suggests that the replication systems of these four plasmids were evolutionarily related to one another.

### Distinct PromA group plasmids are transferred to different bacteria in microbial communities

To assess whether the transconjugant ranges were different in the four PromA plasmids with different nucleotide compositions (GC content and *k*-mer profile), filter mating assays were performed with the donor *P. putida* SMDBS harbouring each plasmid and three different mating conditions (Soil-LBAgar, Soil-Agar, and Lake-Sanaru-LBAgar) as described in the Methods. Transconjugants were identified using the CD (culture-dependent) and CI (culture-independent) methods as described in the Methods. The presence of plasmid DNA in the transconjugants obtained by the CD method was confirmed by PCR. Moreover, the number of transconjugants can be obtained by the CI method; thus, the bacterial taxa of transconjugants of different plasmids were compared.

#### Transconjugants obtained using culture-dependent (CD) methods

Confirmation of plasmid presence and sequencing of the 16S rRNA gene were performed for the sorted 192 colonies per plasmid. Accordingly, 116, 136, 59, and 171 strains for pSN1104-11 (PromAγ, Cluster A), pMH0613-68 (PromAβ−1, Cluster D), pYK0414-12 (PromAβ−2, Cluster C) and pSN0729-62 (PromAδ, Cluster B), respectively, were identified as transconjugants (plasmid positive strains). Different transconjugants of each plasmid were obtained and classified into four phyla, *Proteobacteria* (classes *Alphaproteobacteria *, *Betaproteobacteria *, and *Gammaproteobacteria *), *Firmicutes* (class *Bacilli *), *Bacteroidetes* (classes *Flavobacteriia* and *Sphingobacteriia *), and *Actinobacteria* (class *Actinomycetia *), including 36 genera (Table 2). Bacterial strains belonging to more than two different classes were obtained as transconjugants of any of the four PromA plasmids, suggesting that these plasmids have a broad host range, and some of the taxonomic groups of transconjugants (at the genus and family levels) overlapped with those in previous reports [16, 29, 30]. Six genera (* Stenotrophomonas *, *Enterobacter *, *Klebsiella *, *Raoultella *, *Pseudomonas *, and *Aeromonas *) in *Gammaproteobacteria* were common (shown in red in Table 2) among the 36 genera. Eight of the nine transconjugants belonging to *Alphaproteobacteria* were obtained with pSN1104-11 from all environmental samples, whereas no transconjugants with pMH0613-68 or pYK0414-12 in the class were obtained (Table 2). In contrast, 54 of the 84 transconjugants of pMH0613-68 with SoilLBA were obtained from the class *Betaproteobacteria* (* Comamonas *) (Table 2). Thus, the bacterial taxa of transconjugants of the PromA plasmid with different nucleotide compositions were different.

**Table 2.** Transconjugants of the four PromA plasmids obtained using the culture-dependent method*

Phylum†	Class	Family	Genus	GC%	pSN1104-11 (GC 63.7%)			pMH0613-68 (GC 60.7%)			pYK0414012 (GC 56.5%)			pSN0729-62 (GC 54.2%)		
					SL	SA	LL	SL	SA	LL	SL	SA	LL	SL	SA	LL
	* Alphaproteobacteria* (9)	* Rhizobiaceae* (9)	* Rhizobium* (3)	58–62	1	–	1	–	–	–	–	–	–	–	–	1
			* Agrobacterium* (1)	59	–	1	–	–	–	–	–	–	–	–	–	–
			*Bejierinvkia* (1)	57	1	–	–	–	–	–	–	–	–	–	–	–
			* Ensifer* (4)	62–63	1	3	–	–	–	–	–	–	–	–	–	–
* Proteobacteria* (475)	* Betaproteobacteria* (80)	* Comamonadaceae* (62)	* Comamonas* (60)	60~68	–	–	–	54	–	–	–	–	–	6	–	–
			* Variovorax* (2)	68	–	2	–	–	–	–	–	–	–	–	–	–
		* Burkholderiaceae* (6)	* Burkholderia* (2)	67–68	–	–	–	–	1	–	–	–	–	–	1	–
			* Delftia* (4)	66–67	2	–	–	–	–	–	–	–	–	2	–	–
		* Alcaligenaceae* (12)	* Achromobacter* (12)	64–68	–	1	3	–	–	–	2	–	1	3	–	2
	* Gammaproteobacteria* (386)	* Xanthomonadaceae* (30)	* Aquimonas* (1)	–	–	–	–	–	–	–	–	–	–	–	1	–
			*Stenotrophomonas* (29)	67–68	3	2	–	–	2	–	2	12	2	–	4	2
		* Enterobacteriaceae* (134)	*Enterobacter* (26)	54–57	3	–	–	11	3	3	–	1	–	1	4	–
			*Klebsiella* (17)	53–58	–	9	–	–	5	–	–	1	–	–	1	1
			* Kosakonia* (6)	53–56	4	–	–	2	–	–	–	–	–	–	–	–
			* Lelliottia* (1)	53–56	–	–	–	–	–	–	–	–	–	1	–	–
			* Leclercia* (4)	56	–	–	–	2	2	–	–	–	–	–	–	–
			* Siccibacter* (1)	–	–	–	–	–	1	–	–	–	–	–	–	–
			* Pluralibacter* (2)	59	–	–	–	–	1	–	–	–	–	–	1	–
			* Yokenella* (2)	–	2	–	–	–	–	–	–	–	–	–	–	–
			*Raoultella* (63)	55–57	33	1	–	3	3	–	2	–	–	18	3	–
			* Citrobacter* (8)	51–55	–	–	–	1	1	–	–	1	–	–	5	–
			* Buttiauxella* (2)	51	–	–	–	2	–		–	–	–	–	–	–
			* Kluyvera* (2)	53	–	–	–	1	–	1	–	–	–	–	–	–
		* Erwiniaceae* (1)	* Erwinia* (1)	53–55	–	–	–	–	–		–	–	–	1	–	–
		* Hafniaceae* (1)	* Hafnia* (1)	49	–	–	–	–	–	1	–	–	–	–	–	–
		* Pseudomonadaceae* (97)	*Pseudomonas* (97)	57–67	13	3	6	8	1	–	–	2	2	54	6	2
		* Moraxellaceae* (3)	* Acinetobacter* (3)	35–43	–	–	–	–	–	–	–	–	3	–	–	–
		* Aeromonadaceae* (106)	*Aeromonas* (106)	59–62	–	–	18	–	1	25	–	9	16	1	14	22
		* Vibrionaceae* (6)	* Vibrio* (6)	42–51	–	–	–	–	–	–	–	–	1	–	–	5
		* Shewanellaceae* (8)	* Shewanella* (8)	40–55	–	–	–	–	1	–	–	–	–	–	–	7
* Firmicutes* (1)	* Bacilli* (1)	* Bacillaceae* (1)	* Bacillus* (1)	35–47	–	–	–	–	–	–	1	–	–	–	–	–
* Bacteroidetes* (5)	* Flavobacteriia* (4)	* Flavobacteriaceae* (4)	* Flavobacterium* (1)	31–47	–	–	–	–	–	–	–	–		1	–	–
			* Chryseobacterium* (2)	34–42	–	–	1	–	–	–	–	–	1	–	–	–
			* Myroides* (1)	34	–	–		–	–	–	–	–	–	–	–	1
	* Sphingobacteriia* (1)	* Sphingobacteriaceae* (1)	* Sphingobacterium* (1)	37–44	–	–	1	–	–	–	–	–	–	–	–	–
* Actinobacteria* (1)	* Actinomycetia* (1)	* Promicromonosporaceae* (1)	* Krasilnikoviella* (1)	–	–	1	–	–	–	–	–	–	–	–	–	–
			plasmids positive /192		63	23	30	84	22	30	7	26	26	88	40	43

*‘–’ indicates no transconjugants were obtained. Common genera of the transconjugants for the four PromA plasmids are indicated in red. The conjugation conditions are indicated by SL: Soil-LBAgar, SA: Soil-Agar, and LL: Lake-Sanaru-LBAgar.

†The numbers in parentheses indicate the number of obtained transconjugants.

Notably, some transconjugants did not show the PCR product signals of the *repA* gene of the plasmid (plasmid-negative), especially pYK0414-12, even though they were obtained as fluorescent cells (Table S5). In these strains, plasmids might be lost during colony formation. Only 30 % (58/192) of the strains sorted on the LB plates possessed the plasmid pYK0414-12 (plasmid-positive in Table 2), whereas 89 % (170/192) were plasmid-positive for pSN0729-62. These results indicate that the persistence of plasmids could vary from one plasmid to another, suggesting that the capacities of plasmid replication and/or conjugative transfer (replication and transfer host ranges) and plasmid stability in each bacterium and/or between bacteria could be important factors affecting the behaviour of plasmids in microbial communities.

#### Transconjugants obtained using culture-independent (CI) methods

**Fig. 3.**
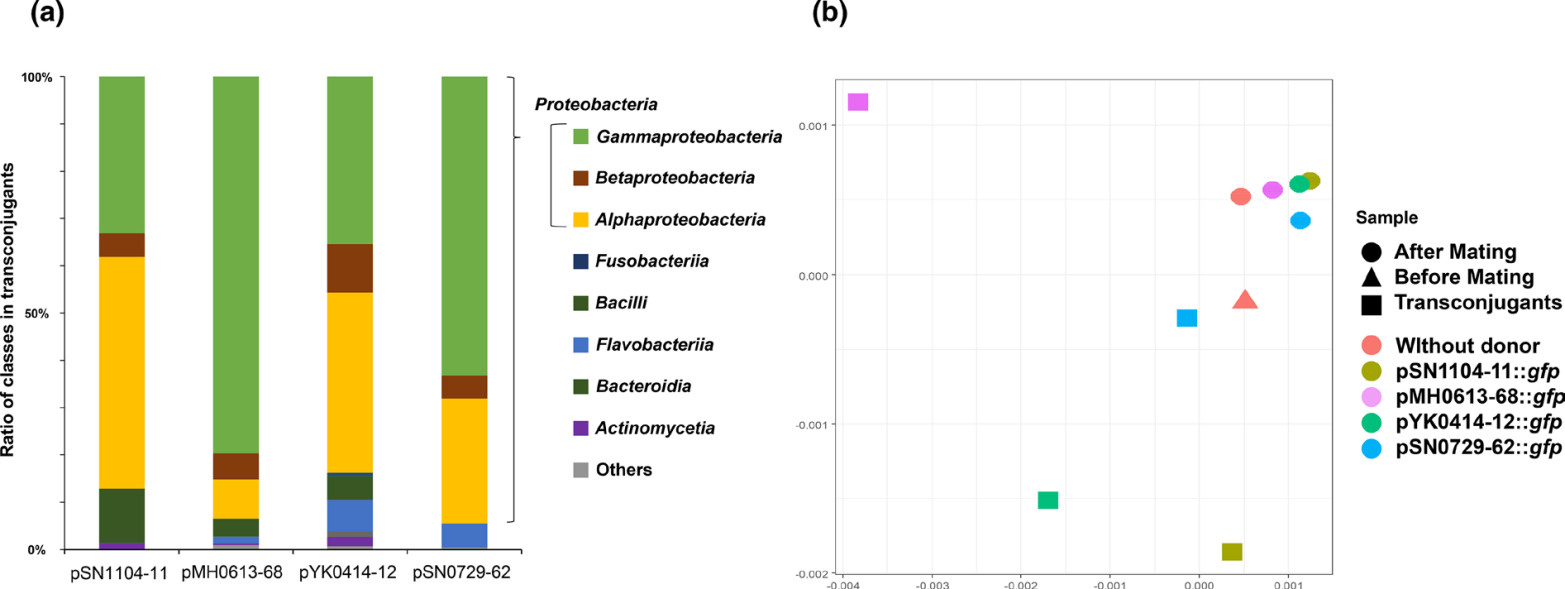
(**a**) Microbial communities of the isolated transconjugants by culture-independent methods from the samples of Lake-Sanaru-LBAgar. (**b**) Principal coordinate analysis (PCoA) plot based on weighted UniFrac distances of microbial communities in mating assays with four PromA plasmids.

To comprehensively compare the range of transconjugants, the sorted transconjugant cells and other samples (before and after mating, Fig. 1) were directly subjected to 16S rRNA gene sequencing. Transconjugants of the four PromA plasmids obtained by the CI method were classified into 12 phyla (* Acidobacteria *, *Actinobacteria, Bacteroidetes, Chlamydiae, Chloroflexi, Cyanobacteria, Firmicutes*, *Fusobacteriia, Gemmatimonadetes, Planctomycetes, Proteobacteria*, and *Verrucomicrobia *, Table S6). The major phylum of the transconjugants was *Proteobacteria* (Figs 3a, S3, S4A), and *Gammaproteobacteria* and *Alphaproteobacteria* were major classes of the transconjugants of the four plasmids in the three mating conditions (Figs 3a and S4A). Notably, the transconjugant class ratios were different; those of *Alphaproteobacteria* were higher in the two plasmids, pSN1104-11 (49 %) and pYK0414-12 (38 %), than those of the other two plasmids, pMH0613-68 (8 %) and pSN0729-62 (26 %) (Fig. 3A, transconjugants obtained from Lake Sanaru under LBAgar conditions). Conversely, the *Gammaproteobacteria* ratios were higher in pMH0613-68 (80 %) and pSN-729–62 (63 %) than in pSN1104-11 (33 %) and pYK0414-12 (36 %) (Fig. 3a). A similar tendency was observed in the other two mating conditions (Fig. S4), even though their recipient communities were not similar at the family level (Fig. S5). A more detailed analysis of the transconjugants revealed that some were recognized as different operational taxonomic units even though they belong to the same genus (Fig. S6). Notably, some in different operational taxonomic units but belonging to the same genus obtained different PromA plasmids (Fig. S6). This indicates that transconjugant ranges of PromA plasmids might vary at the level of bacterial species and strains with different nucleotide compositions.

The microbial communities of the three sample types described in the Methods (Fig. 1) were compared, and PCoA plots of the weighted UniFrac distance for the microbial communities are shown in Fig. 3b. The results revealed that the distances between the fraction of ‘After Mating’ were short regardless of the presence or absence of the donor, which accounted for half of the bacterial communities on filter mating. In contrast, the distances among the plots of ‘Transconjugants’ were considerably larger than those of ‘After Mating’, suggesting that transconjugant bacterial communities of each PromA plasmid were different. Collectively, the results of both the CD and CI methods indicate that the transconjugant bacterial taxa (i.e. bacterial hosts in which the plasmid has been transferred to or replicated in) of four PromA plasmids with different nucleotide compositions are different.

### PromA plasmids show different degrees of similarity in nucleotide compositions to bacterial chromosomes

**Fig. 4.**
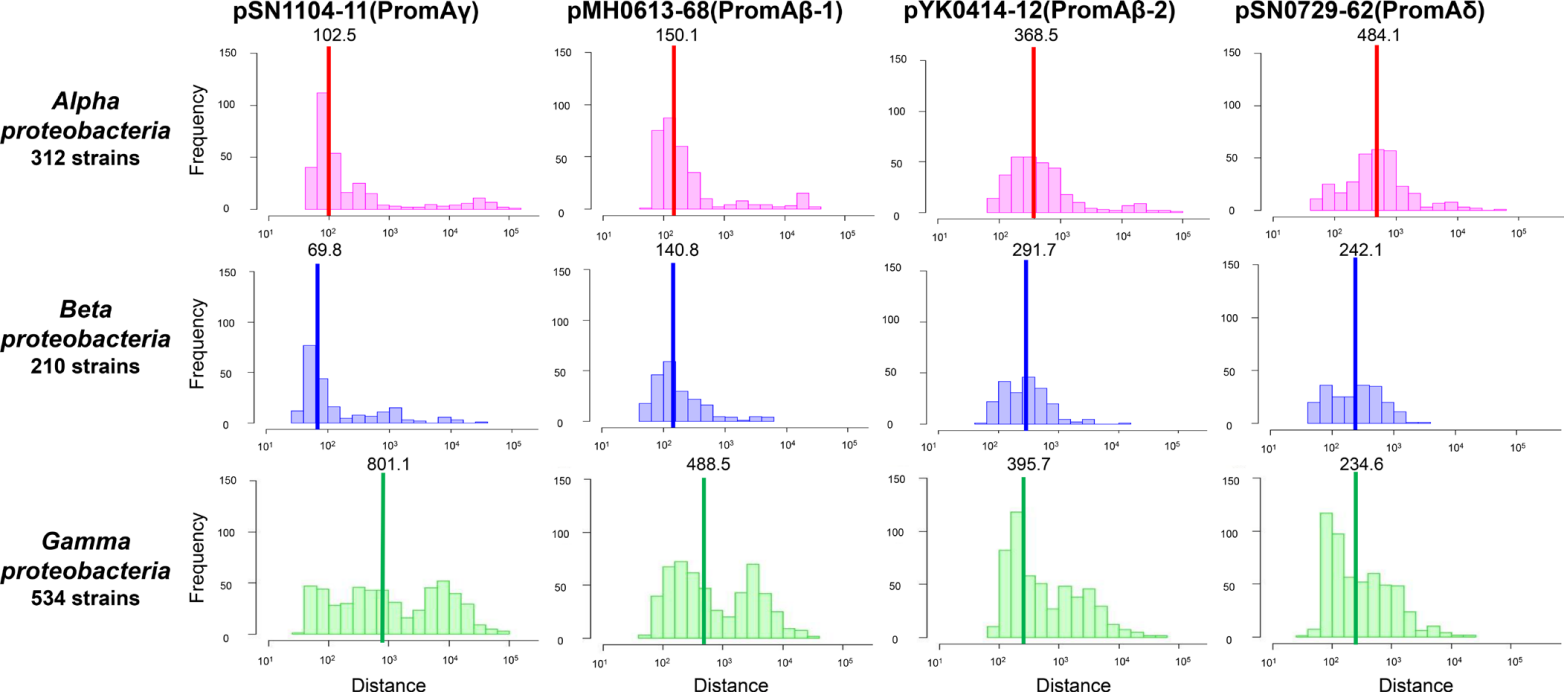
Distribution of Mahalanobis distances of 3-mer compositions between plasmids and chromosomes of *Alpha-, Beta-,* and *Gammaproteobacteria *. Bold lines in each histogram indicate the median value of the Mahalanobis distance.

The dissimilarity (Mahalanobis distances, *D*) of *k*-mer (*k*=2, 3, 4) compositions was calculated between each PromA plasmid and 2772 reference genomes (Table S7). The distributions of *D* ^2^ of the 3-mer composition are shown in Fig. 4 for the three classes (*Alpha-, Beta-,* and *Gammaproteobacteria *) of *Proteobacteria *, the major obtained transconjugant classes. Interestingly, the distribution was altered not only by different plasmids but also by different bacterial classes (Fig. 4). For pSN1104-11 (PromAγ, Cluster A) and pMH0613-68 (PromAβ−1, Cluster D), a single peak was observed for *Alpha-* and *Betaproteobacteria* whereas two peaks were observed for *Gammaproteobacteria *. One of the peaks in *Gammaproteobacteria *, showing shorter distances, included *Pseudomonadaceae* and *Enterobacteriaceae,* whereas the other included *Vibrionaceae, Pasteurellaceae,* and *Moraxellaceae* (Table S7). For pYK0414-12 (PromAβ−2, Cluster C) and pSN0729-62 (PromAδ, Cluster B), only a single peak was observed in all three classes (Fig. 4). The median *D* ^2^ for each of the three classes of *Proteobacteria* varied among the four PromA plasmids (Fig. 4), suggesting different evolutionary host ranges.

Previously, it was proposed that a bacterial strain could be predicted as a candidate for the evolutionary host of plasmids if the *P*-value of *D* ^2^ is larger than 0.6 [19]. For example, the F plasmid showed a high *P*-value with *E. coli *, the representative hosts of this plasmid [19]. Although this threshold worked well for narrow-host-range plasmids including F, no evolutionary host candidates were detected for broad-host-range plasmids including IncW, IncP/P-1, and PromA [19]. Here, no bacterial chromosomes showed high *P*-values for our PromA plasmids, and the maximum *P*-values were 0.316 for pSN1104-11, 0.140 for pMH0613-68, 0.076 for pYK0414-12, and 0.179 for pSN0729-62. Even for the known host (genera) of PromA plasmids (PromAβ−1: *Collimonas* and *Thiomonas *; PromAγ: *Hydrogenophaga* and *Variovorax *), the *P*-value (0.007–0.136) was considerably smaller than 0.6. These results indicate that the evolutionary host candidates of each PromA plasmid were not clearly predicted in the 2772 reference bacteria and that PromA plasmids probably showed a broad host range and were not hosted in a narrow/limited range of bacteria. Notably, the maximum *P*-value of pYK0414-12 was considerably lower than that of the other three plasmids implying that evolutionary hosts with high plasmid *P*-values might not exist in the 2772 reference bacteria. This is potentially related to the fact that the number of plasmid positive strains of pYK0414-12 was lower than that of other PromA plasmids in the CD method (Table 2).

### The similarity of *k*-mer plasmid compositions and transconjugant chromosomes compared to those of other bacterial chromosomes

**Fig. 5.**
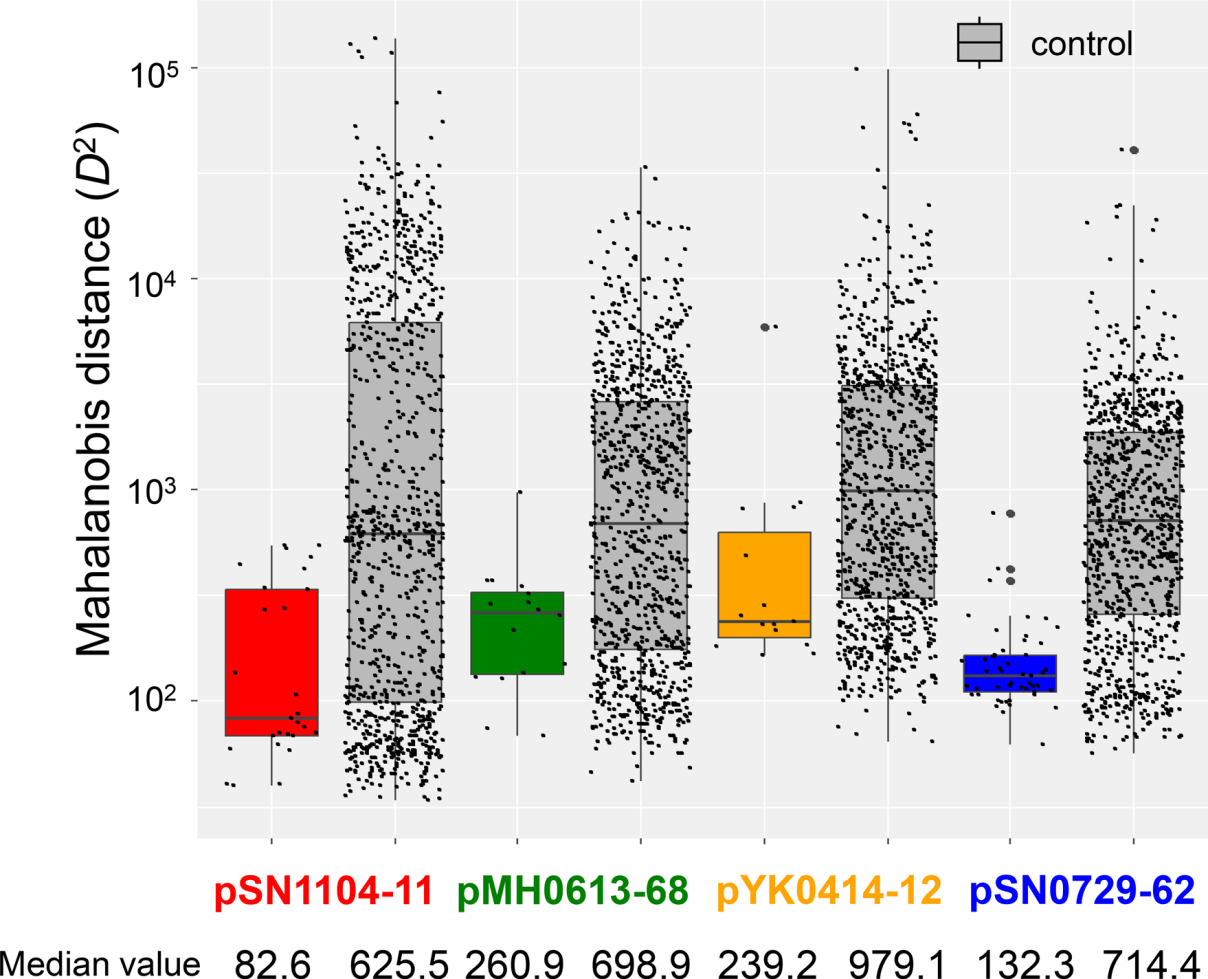
Box plots of Mahalanobis distances of 3-mer between each PromA plasmid and its transconjugants (coloured) and between the plasmid and 965 recipient reference genomes (grey). A median value in each data is shown by a black line in each box.

To assess whether the *k*-mer compositions of the plasmids and transconjugants were similar, the draft genome sequences of 107 transconjugants obtained by the CD method were determined (27 for pSN1104-11, 16 for pMH0613-68, 15 for pYK0414-12, and 49 for pSN0729-62) (Table S8). They were classified into three classes, 13 families, and 21 genera (Table S8). The *D* ^2^ distribution of the *k*-mer compositions is shown in Fig. 5 (*k*=3) and S7 (*k*=2, 4). Their *D* ^2^ values were compared to those of the 965 genomes selected from the 2772 reference genomes (Figs 5 and S6). The 965 genomes were selected by using microbial community data in different environmental samples including soil and lake water (shown in red in Table S7). The *D* ^2^ of the 3-mer compositions between plasmids and chromosomes of the transconjugants (coloured) was lower than the median of those between plasmids and chromosomes of the above 965 genomes (grey, 625.5 for pSN1104-11, 698.9 for pMH0613-68, 979.1 for pYK0414-12, and 714.4 for pSN0729-62) (Fig. 5). There were significant differences in *D* ^2^ between PromA plasmids and bacterial chromosomes (*P*=1.47×10^−5^ for pSN1104-11, *P*=4.35×10^−3^ for pMH0613-68, *P*=4.32×10^−3^ for pYK0414-12, and *P*=2.20×10^−16^ for pSN0729-62). Considering the taxonomic bias of the 107 transconjugants, bacterial genomes of the same genus as the transconjugants, identified by the CD method, were selected and compared for their *D^2^* value (Fig. S8). In addition to the 107 transconjugants, those in the same genus were more similar to the plasmid than other recipient reference genomes (Fig. S8). Notably, the number of strains of the same genus in the 2772 reference genomes was not uniform, and the composition of *k*-mer varied even within the same genus (Fig. S9). Thus, the number and composition of bacteria of the same genus in the reference genomes would significantly affect the results. Nevertheless, in some cases, there were clear differences in *k*-mer compositions between the plasmid and the host or non-host chromosomes. Using either CD or CI methods, *Shewanella* strains were obtained as transconjugants of pSN0729-62 and pMH0613-68, but not of pSN1104-11. The *D* ^2^ (3-mer) between pSN1104-11 and pSN0729-62, and the *Shewanella* chromosome, was larger than 3000 and 200, respectively (Table S8). Similarly, *Citrobacter* strains showed similar *k*-mer compositions to pSN0729-62 (116.7–118.0 for 3-mer) but dissimilar *k*-mer compositions to pSN1104-11 (1450.8~1471.1 for 3-mer) (Table S8). Only three of the 107 transconjugants, *Hafnia* (pMH0613-68), *Acinetobacter* (pYK0414-12), and *Stenotrophomonas* (pSN0729-62), showed a larger *D* ^2^ than the median of the reference genomes (Table S8). These results indicate that the *k*-mer compositions of the plasmid were similar to those of the chromosomes of the experimentally obtained transconjugants and that this similarity could be partially used to predict the transconjugant range of plasmids.

The *k*-mer compositions between each transconjugants were compared. The 3-mer of 107 transconjugants were calculated and their clusters are shown in Fig. S10. The 3-mer clusters were well-coincided with the phylogeny of these transconjugants (Fig. S10). Notably, for transconjugants belonging to the same genus *Pseudomonas *, there were three different clusters with different PromA plasmids (Fig. S10), indicating that the different plasmids had truly different host distributions due to their nucleotide composition differences. With respect to the phylogenetic analyses of transconjugants obtained by the CI method, again, the distribution of transconjugants was different based on the plasmid (Fig. S6). Next, all genomic data for all transconjugants were collected and the Mahalanobis distance between each PromA plasmid and its transconjugants, and the non-transconjugants among them, were compared (Fig. S11). As a result, pSN1104-11 (PromAγ, cluster A) or pSN0729-62 (PromAδ, cluster B) – having the most different GC content (64 % for pSN1104-11 and 54 % for pSN0729-62) – showed a higher similarity to its transconjugants than to the non-transconjugants (those of other PromA plasmids) (Fig. S11). Additionally, the GC content of transconjugants obtained by the CD method was similar to that of the plasmids (Table 2), while there was no significant difference between plasmids, indicating that *k*-mer composition is more suitable than GC content for plasmid host prediction.

## Conclusion

The evolutionary host range of plasmids can be predicted based on the nucleotide sequence of the plasmids and bacterial chromosomes because host-specific mutation bias may homogenize the nucleotide compositions of plasmid host chromosomes in which the plasmid has remained for a long time, referred to as amelioration [18, 19, 52]. However, this evolutionary host range is unknown and experimentally undeterminable. Here, different transconjugant ranges were identified among four PromA plasmids with different nucleotide compositions (Table 2, Figs 2 and 3), indicating that the four plasmids may have different host ranges. The similarity of nucleotide compositions between the chromosomes of transconjugants and their plasmids was higher than that between the chromosomes of most reference bacteria and plasmids. This is probably because the similarity is important for the replication and maintenance of plasmids and/or the expression of genes. Plasmid replication and maintenance is usually dependant on the host factors including DnaA, DNA polymerases, helicase and single strand DNA binding proteins, and others. The interactions between these factors and plasmid DNA could be influenced by the plasmid nucleotide compositions. Codon usage is also an important factor for efficient expression of genes related to the replication, maintenance, and conjugative transfer of plasmids. Indeed, some proteins involved in these functions, including RepA, KorB, and Tra proteins, showed a low identity (<60 %) at their amino acid sequence levels (Table S3). In addition, several transconjugants obtained by the CD method lost their plasmids during colony formation, suggesting that the similarity might have a stronger influence on long-term plasmid behaviour. Further studies are required for accurate predictions, including (i) their replication host ranges using mini replicons; (ii) their transfer host ranges using the plasmids carrying the same replicon system (*repA* and *oriV*) and different transfer systems; (iii) mating assays with mock bacterial communities composed of bacterial strains with available genome sequences; and (iv) comparisons of plasmid persistence in different hosts with different nucleotide compositions. Obviously, the similarity of the nucleotide compositions is not the only factor used to determine the plasmid host. It should also be considered that the hosts possessed exclusive systems to defend themselves from the external DNA elements, including restriction and modification systems, CRISPR-Cas systems, and/or entry/surface exclusion systems of the ‘indigenous’ plasmid [53, 54]. These studies will provide an important clue for tracking gene transfer caused by plasmid conjugation in nature.

## Supplementary Data

Supplementary material 1

Supplementary material 2
