# Supplementary material for: Transconjugant range of PromA plasmids in microbial communities is predicted by sequence similarity with the bacterial host chromosome
Source: Microb Genom. 2023 Jun 21;9(6):mgen001043. doi: 10.1099/mgen.0.001043 (PMC10327500; doi:10.1099/mgen.0.001043)
Supplement: Supplementary material 1 [file mgen-9-1043-s001.pdf]

Supplemental materials

Transconjugant range of PromA plasmids in microbial communities is predicted by sequence similarity with the bacterial host chromosome

Tokuda et al.

Correspondence: Masaki Shintani (shintani.masaki@shizuoka.ac.jp), Haruo Suzuki (haruo@sfc.keio.ac.jp)

The Supplementary materials include:

Figures S1-S11

Tables S1-S8 (provided in Excel format)

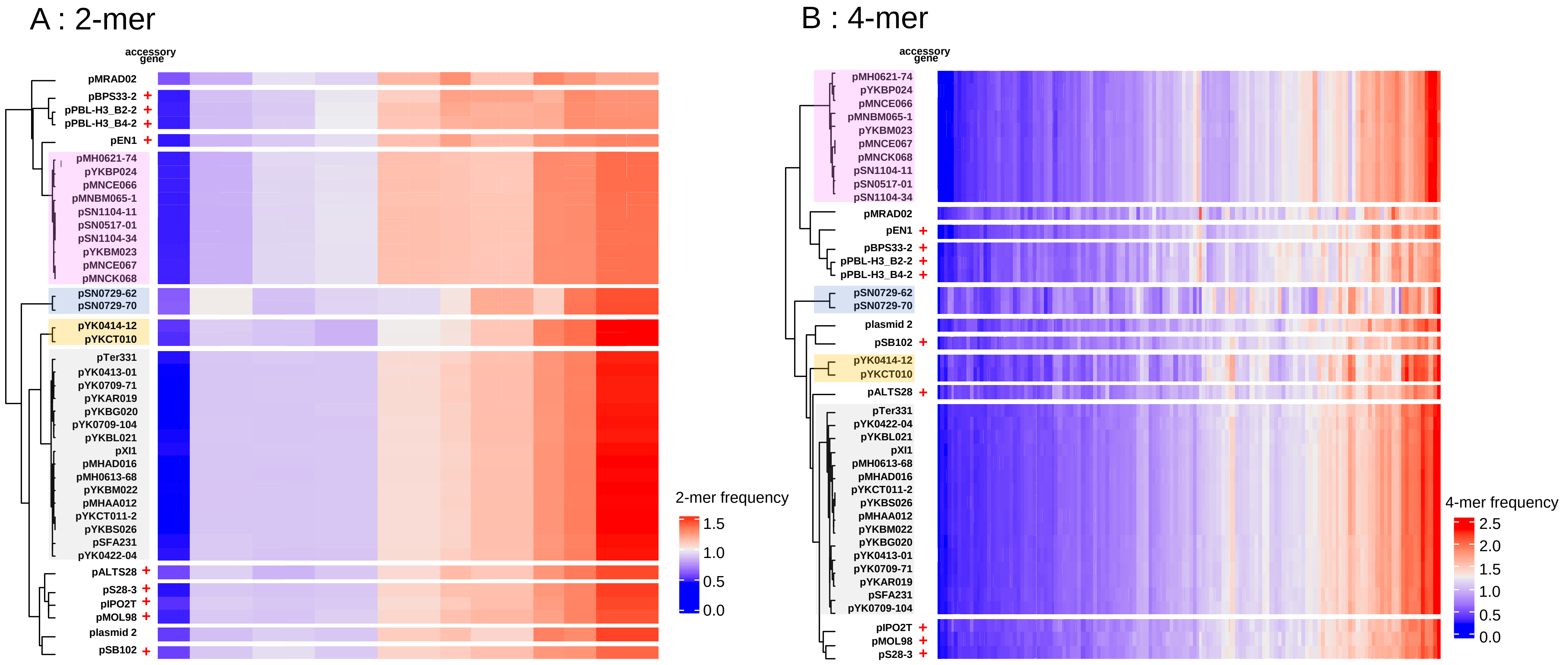

Figure S1. A clustered heatmap showing the dissimilarity of (A) 2-mer (B) 4-mer compositions in PromA plasmids. Dissimilarities were calculated using Euclidean distance. 2,4-mer frequency is the observed 2,4-mer frequency divided by the expected 2,4-mer frequency. Nine plasmids carrying known accessory genes are indicated by “+”.

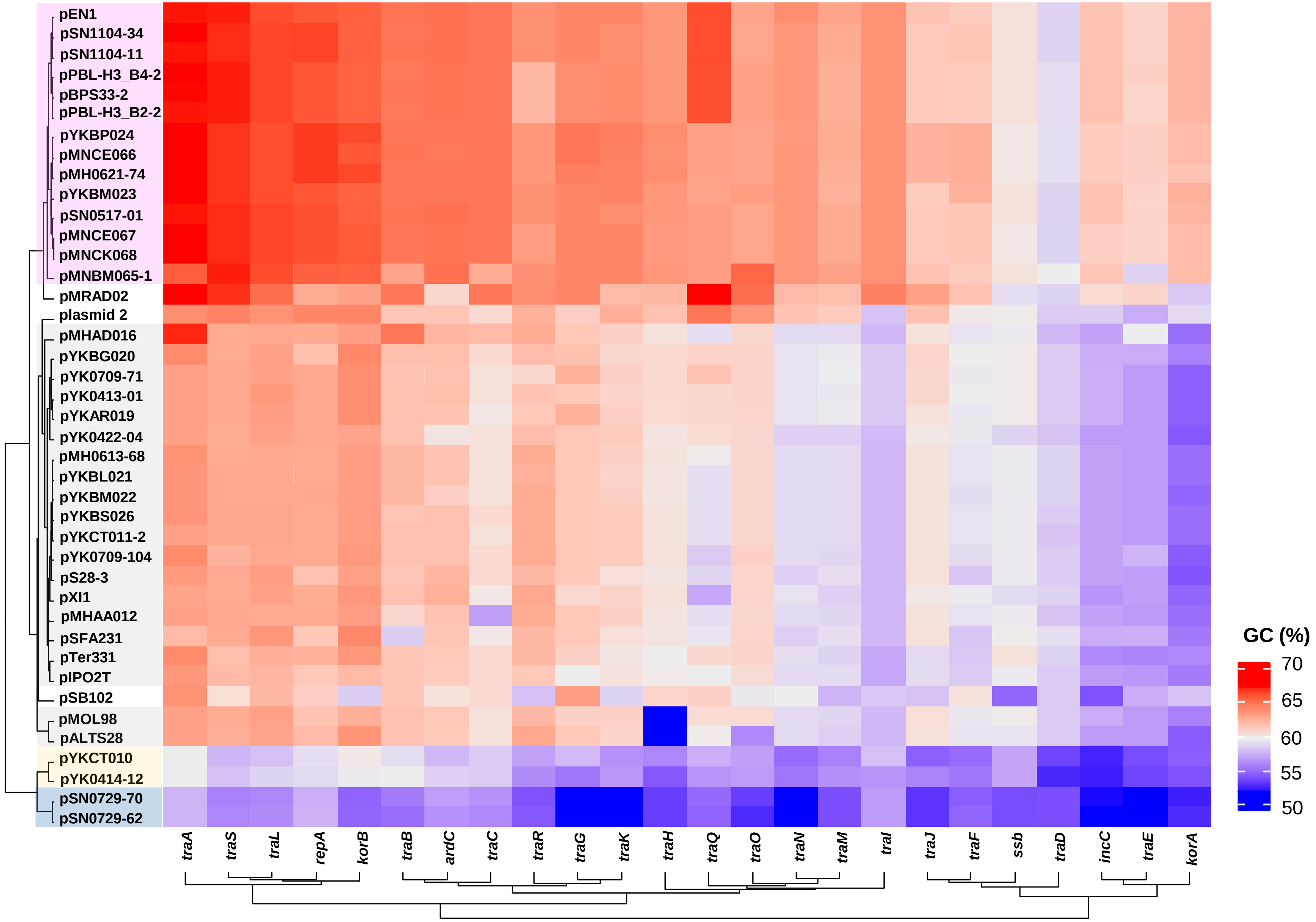

Figure S2. A clustering heatmap showing the GC contents of 24 conserved genes of PromA plasmid based on Table S2. Dissimilarities were calculated using Euclidean distance.

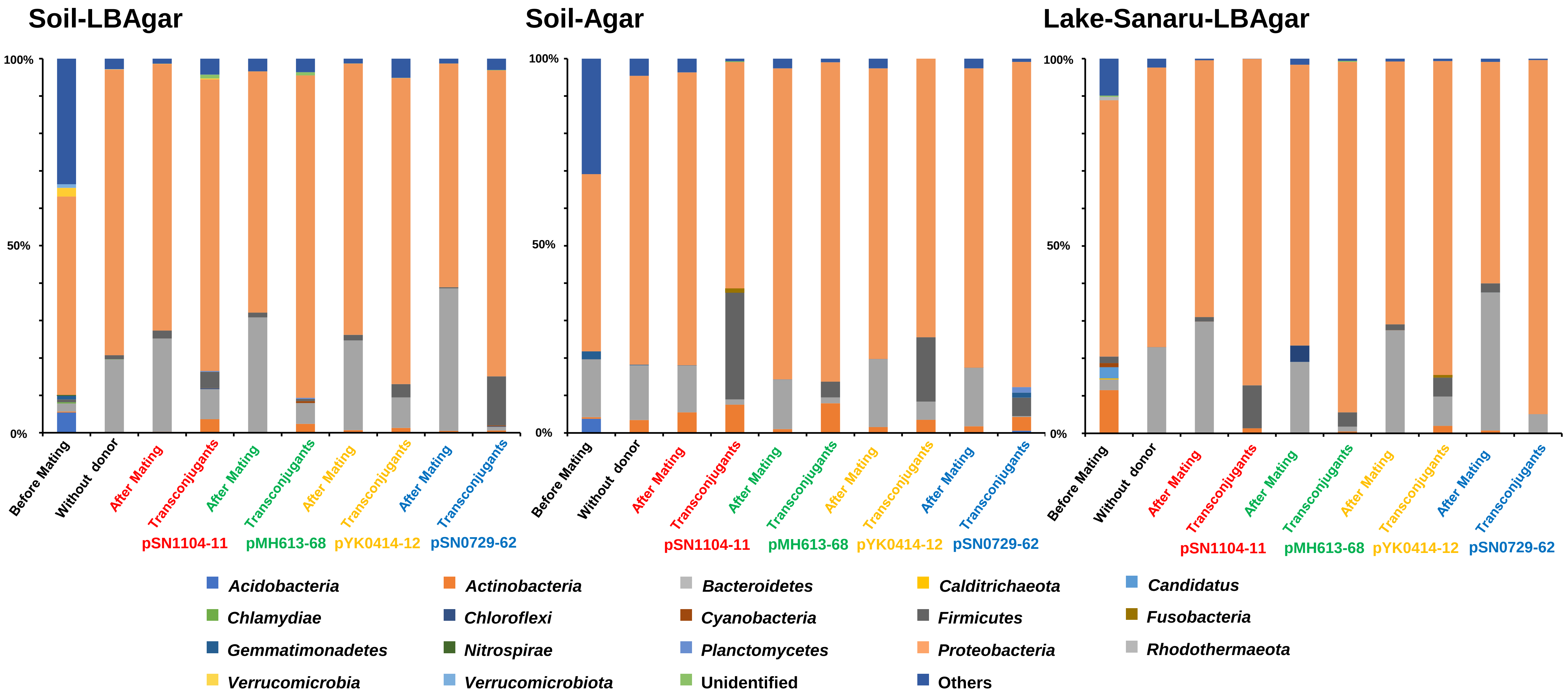

Figure S3. Microbial communities of before and after filter mating and isolated transconjugants in CI methods at phylum level in each condition (Soil-LBAgar, Soil-Agar, Lake-Sanaru-LBAgar).

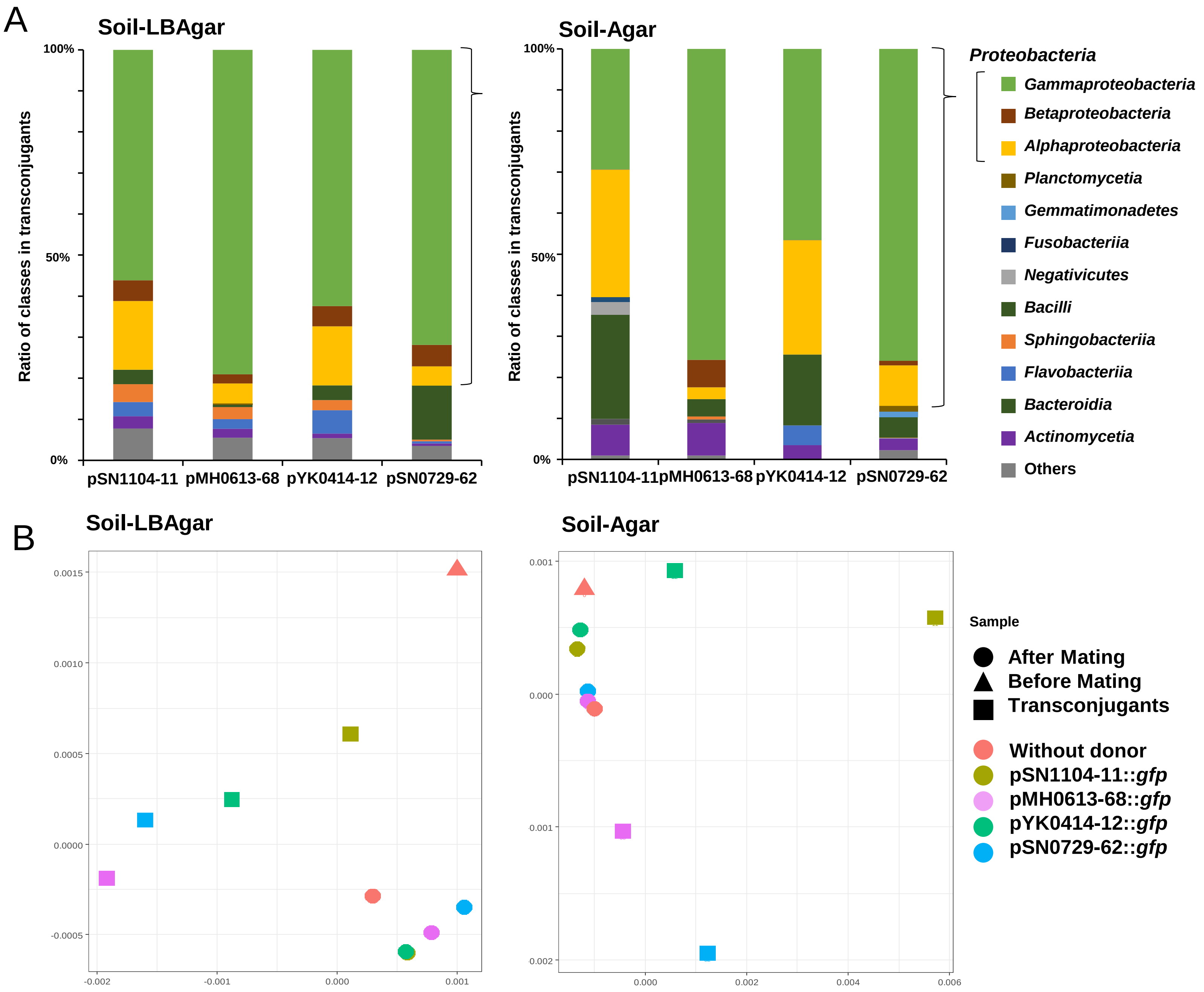

Figure S4. (A) Microbial communities of isolated transconjugants in each condition (Soil-LBAgar, Soil-Agar) at class level in CI method. (B) Principal coordinate analysis (PCoA) plot based on weighted UniFrac distance showing the similarity of microbial communities of before and after conjugation and isolation.

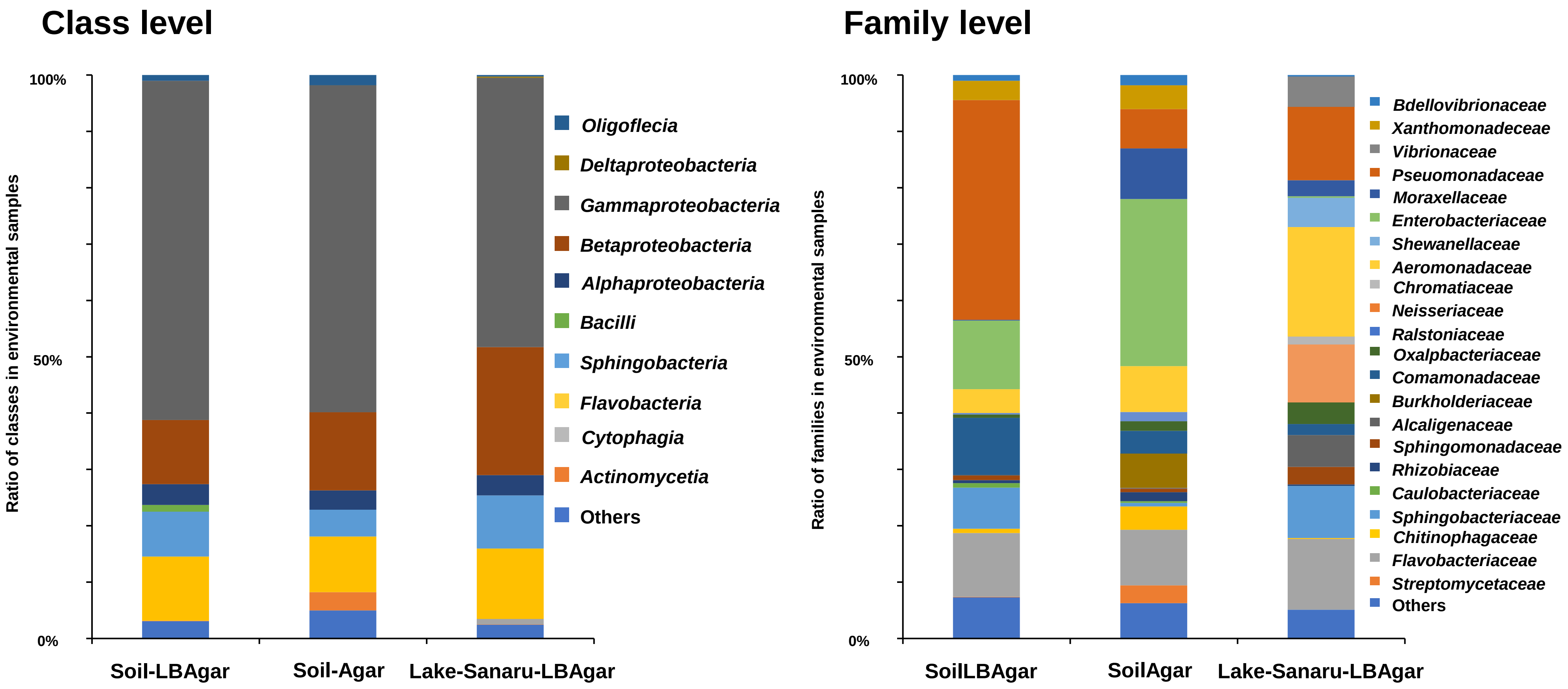

Figure S5. Microbial communities of the recipients (Before Mating sample) of each conjugation condition in class and family level.

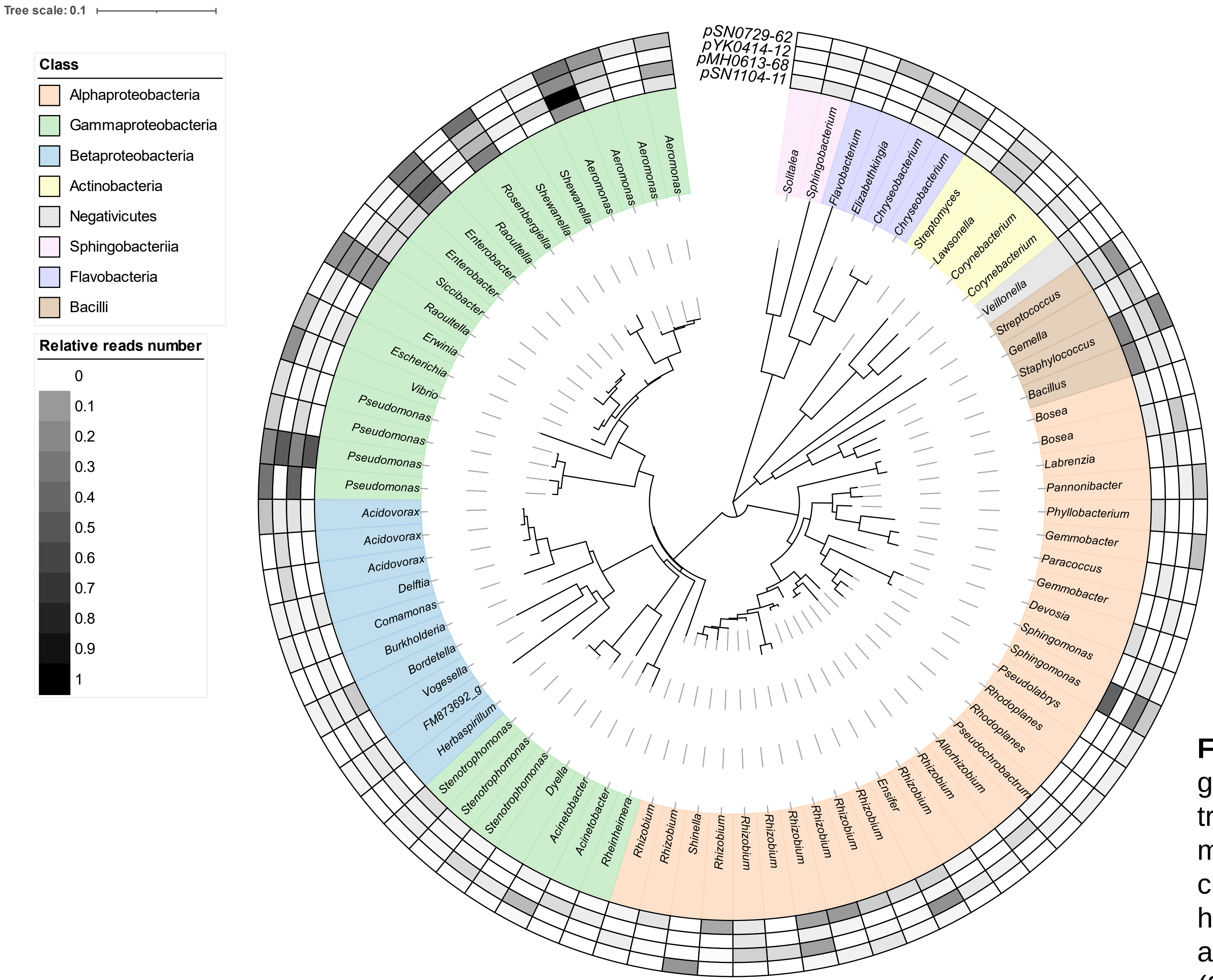

Figure S6. Phylogenetic tree based on 16S rRNA genes showing the genera of top 10% OTUs of transconjugants analyzed by culture-independent method. The colours of genus name indicate the classification at the class level, and the outer heatmap indicates the total read numbers of amplicon sequence of the three mating assays (Soil-LBAgar, Soil-Agar and Lake-Sanaru-LBAgar).

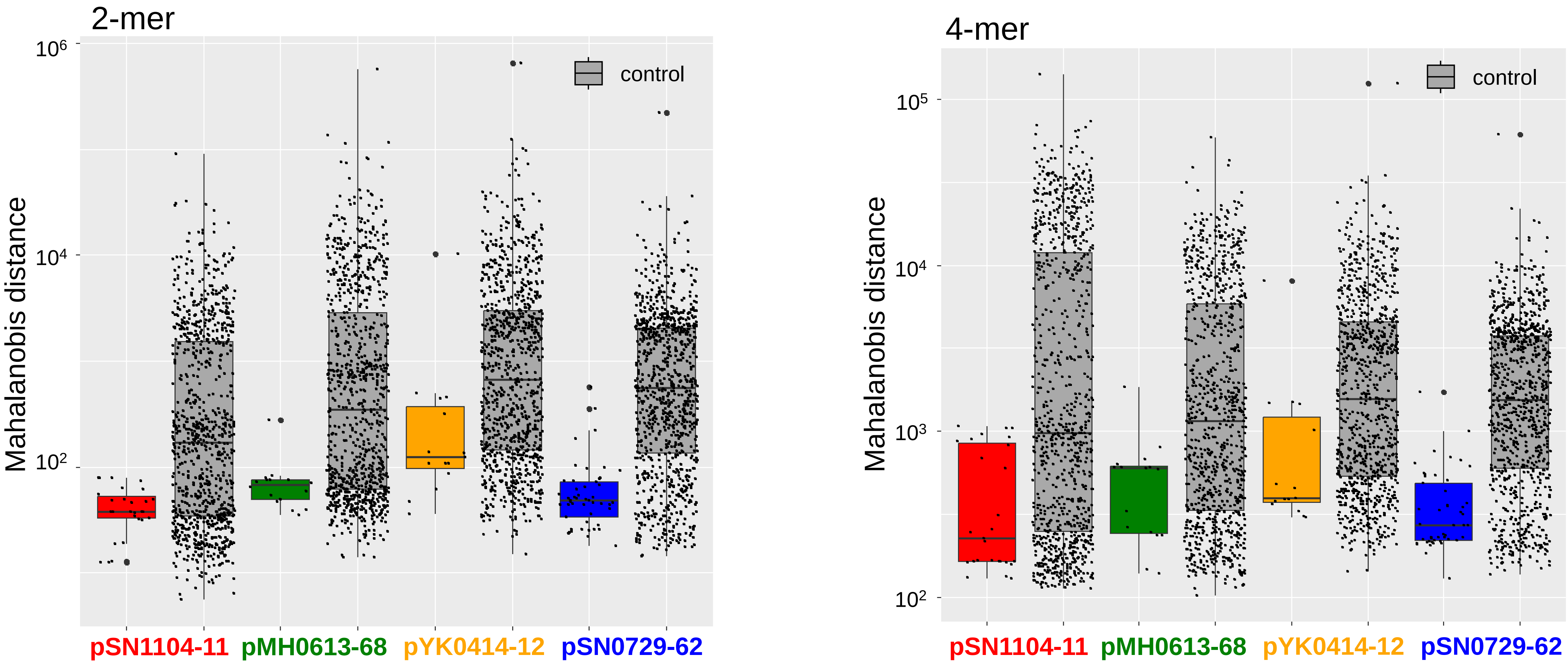

Figure S7. Box plots of Mahalanobis distance of 2,4-mer between each PromA plasmid and its transconjugants (colored) and between the plasmid and reference genomes (gray). A median value in each data is shown by a black line in each box. There were statistical significance between transconjugants and reference genomes of each plasmid ($p < 0.05$,wilcox test).

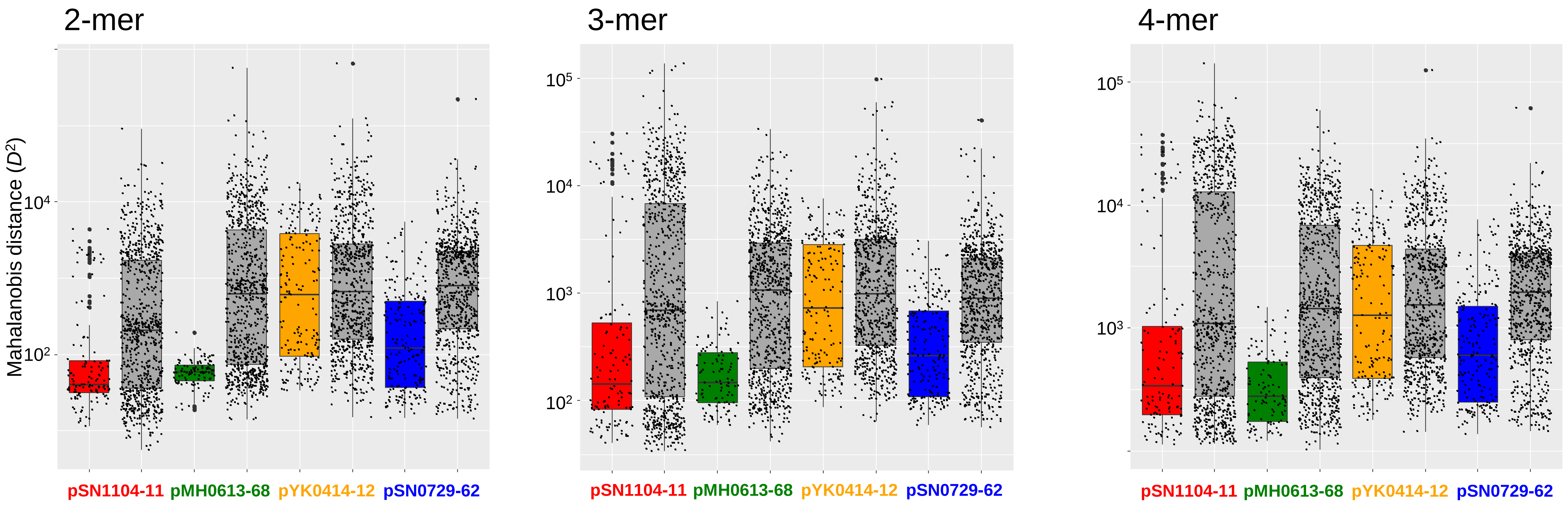

Figure S8. Box plots of Mahalanobis distance of 2,3,4-mer between each PromA plasmid and its same genus strain with transconjugants identified by culture-dependent method (colored) and between the plasmid and reference genomes (gray). A median value in each data is shown by a black line in each box.

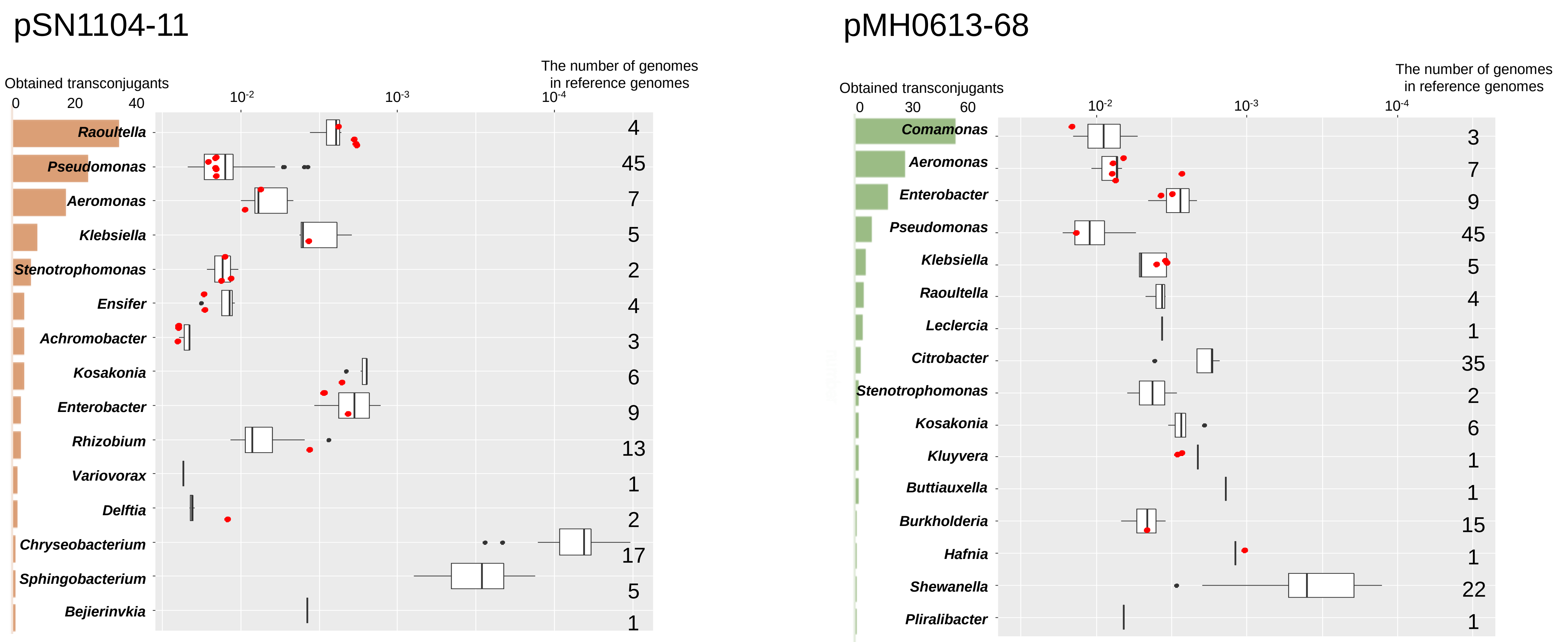

Figure S9. Box plot showing the difference of Mahalanobis distance of 3-mer compositions between the same genus bacteria with transconjugants identified by culture-dependent method. The red plots indicate the distance of sequenced transconjugants (107 strains). The bar plots on the left indicate the amount of obtained transconjugants obtained by CD method, and the numbers of the genus in the reference genomes were shown at right side of the figure.

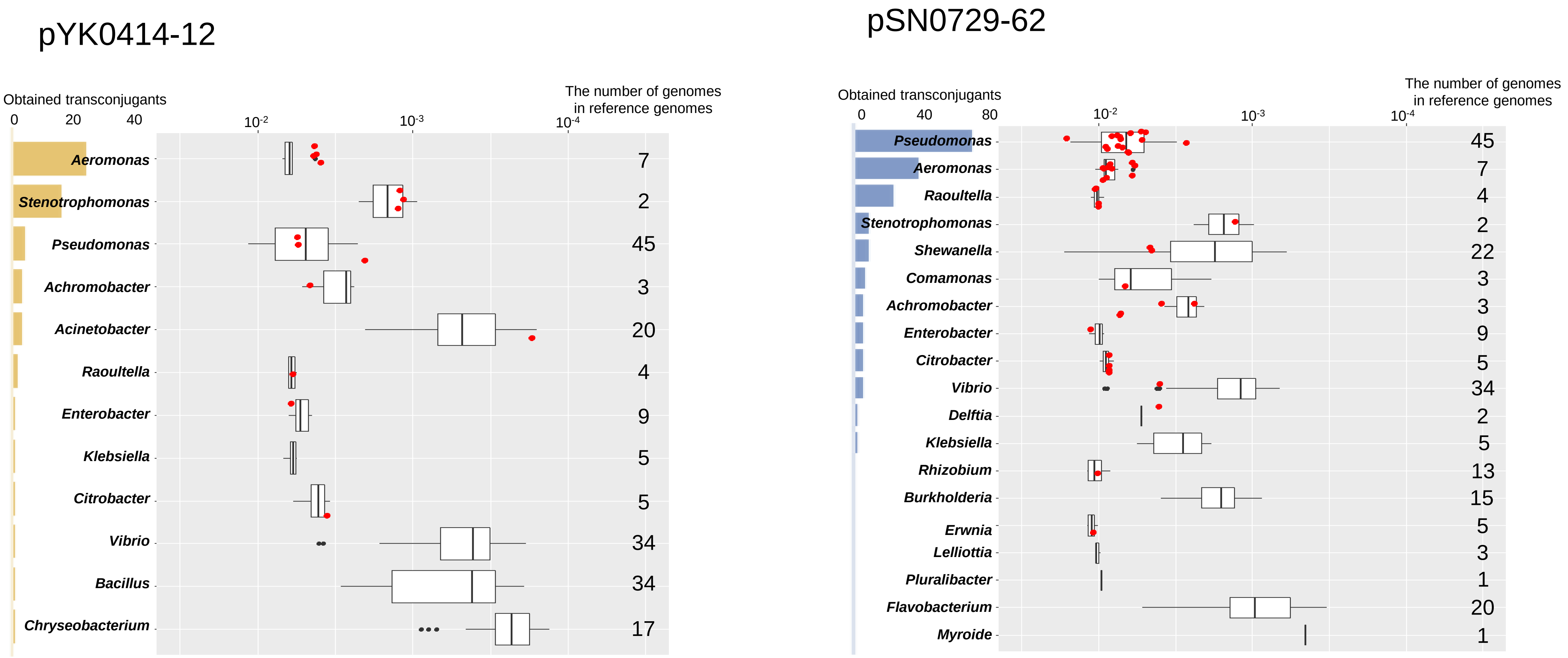

Figure S9. *Continued.*

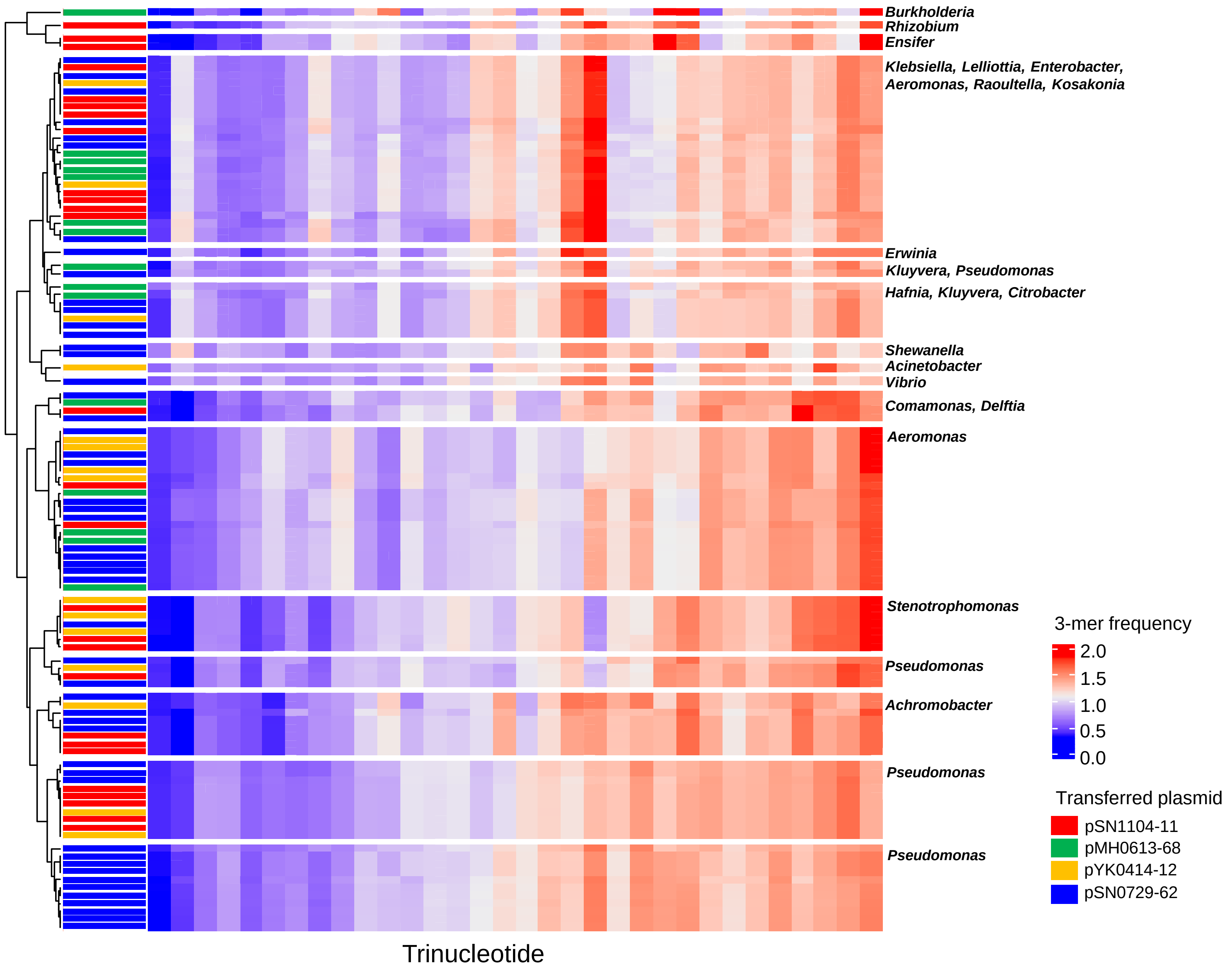

Figure S10. Heatmap clustering the sequenced 107 transconjugants chromosomes based on the dissimilarity of 3-mer compositions. Dissimilarities were calculated using Euclidean distance. 3-mer frequency is the observed 3-mer frequency divided by the expected 3-mer frequency.

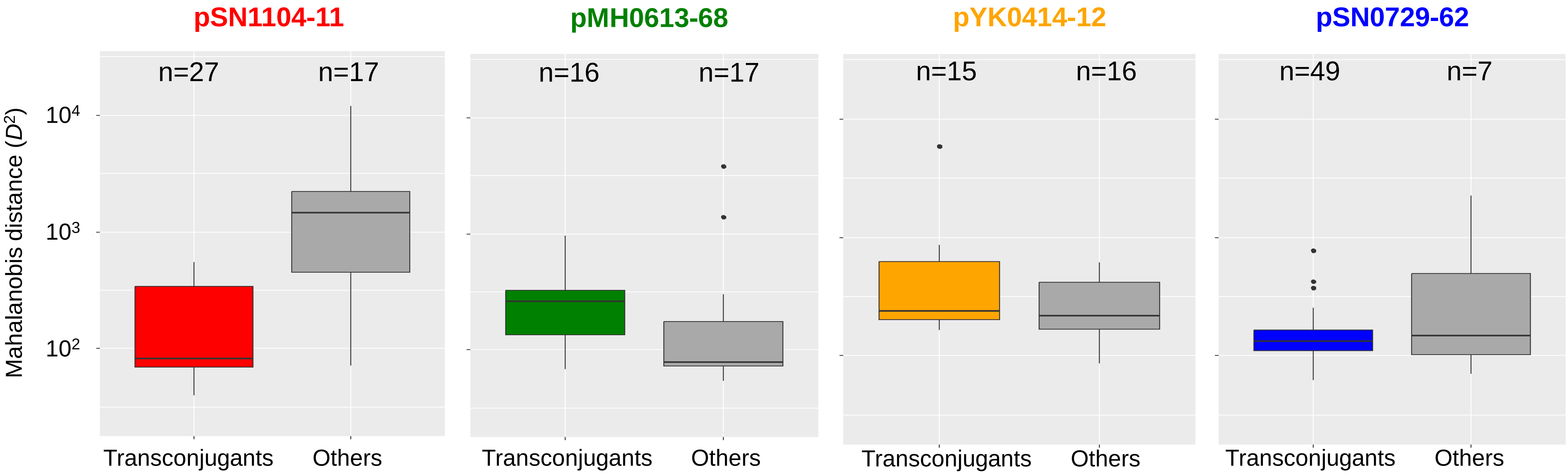

Table S11. The boxplot comparing the Mahalanobis distance of 3-mer compositions between sequenced transconjugants (colour) and non-transconjugants which obtained as other PromA plasmids transconjugants(Others, gray) for each plasmid. A median value in each data is shown by a black line in each box.
